# Designing lipid nanoparticles using a transformer-based neural network

**DOI:** 10.1038/s41565-025-01975-4

**Published:** 2025-08-15

**Authors:** Alvin Chan, Ameya R. Kirtane, Qing Rui Qu, Xisha Huang, Jonathan Woo, Deepak A. Subramanian, Rajib Dey, Rika Semalty, Joshua D. Bernstock, Taksim Ahmed, Rowan Honeywell, Charles Hanhurst, Isaac Diaz Becdach, Leah S. Prizant, Ashley K. Brown, Hao Song, Justin Law Cobb, Louis B. DeRidder, Bruna Santos, Miguel Jimenez, Michelle Sun, Yuebin Huang, Ceara Byrne, Giovanni Traverso

**Affiliations:** 1 Department of Mechanical Engineering, Massachusetts Institute of Technology, Cambridge, MA USA; 2 Division of Gastroenterology, Hepatology and Endoscopy, Department of Medicine, Brigham and Women’s Hospital, Harvard Medical School, Boston, MA USA; 3 David H. Koch Institute for Integrative Cancer Research, Massachusetts Institute of Technology, Cambridge, MA USA; 4 College of Computing and Data Science, Nanyang Technological University, Singapore, Singapore; 5 Lee Kong Chian School of Medicine, Nanyang Technological University, Singapore, Singapore; 6 Department of Pharmaceutics, University of Minnesota, Minneapolis, MN USA; 7 Masonic Cancer Center, University of Minnesota, Minneapolis, MN USA; 8 University of Toronto, Toronto, Ontario Canada; 9 Broad Institute of MIT and Harvard, Cambridge, MA USA; 10 Department of Neurosurgery, Brigham and Women’s Hospital, Harvard Medical School, Boston, MA USA; 11 Northeastern University, Boston, MA USA; 12 Australian Institute for Bioengineering and Nanotechnology, The University of Queensland, Brisbane, Queensland Australia; 13 Harvard-MIT Division of Health Science Technology, Massachusetts Institute of Technology, Cambridge, MA USA; 14 Department of Biomedical Engineering, Boston University, Boston, MA USA; 15 Present Address: Broad Institute of MIT and Harvard, Cambridge, MA USA

**Keywords:** Nanoparticles, Nanoparticles, Nanoparticles, Drug delivery

## Abstract

The RNA medicine revolution has been spurred by lipid nanoparticles (LNPs). The effectiveness of an LNP is determined by its lipid components and their ratios; however, experimental optimization is laborious and does not explore the full design space. Computational approaches such as deep learning can be greatly beneficial, but the composite nature of LNPs limits the effectiveness of existing single molecule-based algorithms to LNPs. Addressing this, our approach integrates the multi-component and multimodal features of composite formulations such as LNPs to predict their performance in an end-to-end manner. Here we generate one of the largest LNP datasets (LANCE) by varying LNP formulations to train our deep learning model, COMET. This transformer-based neural network not only accurately predicts the efficacy of LNPs but is adaptable to non-canonical LNP formulations such as those with two ionizable lipids and polymeric materials. Furthermore, COMET can predict LNP performance in a cell line outside of LANCE and predict LNP stability during lyophilization using only small training datasets. Experimental validation showed that our approach can identify LNPs that exhibit strong protein expression in vitro and in vivo, promising accelerated development of nucleic acid therapies with extensive potential across therapeutic and manufacturing applications.

## Abstract (web summary)

Preventing endosomal damage sensing or using lipids that create reparable endosomal holes reduces inflammation caused by RNA–lipid nanoparticles while enabling high RNA expression.

## Main

For clinical^1^, logistical^2^ and translational^3^ success, most drug substances are formulated into drug products with multiple ingredients. Our analysis shows that, on average, eight excipients are present in commercial products^4^. Given choices of ingredients and their ratios, formulation design presents a vast search space. While high-throughput approaches exist^5–8^, they become intractable with increasing formulation complexity.

Deep learning, a branch of machine learning suited for multifactorial data, can help address this challenge. Although widely used in drug discovery and materials science^9–11^, its application to multi-component drug products is limited. We apply deep learning to RNA-based lipid nanoparticles (LNPs), a promising class of drug products^12–14^, underscored by the success of SARS-CoV-2 messenger RNA vaccines^15–18^. LNPs comprise four lipid classes, each crucial for cytosolic RNA delivery^12,14,19^. Their function depends on lipid structures and ratios^20–22^, with composition requiring re-optimization per application^1,23^.

Given these challenges, early efforts applying machine learning to drug delivery have emerged, including recent work from our group^24^ and others^25^. As lipid chemical structure has a major impact on transfection, a line of modelling approaches focus primarily on individual molecules^25,26^. These approaches have been remarkably successful in identifying new lipids and chemical substructures, which would otherwise not be expected to produce high transfection efficacy^26^. Some models rely on manually selected features such as physicochemical properties^26–29^. These face limitations: restricted LNP scope, underuse of raw data, synthetic feasibility constraints and lack of formulation composition insights. To unlock deep learning’s full utility for LNP design, a model must represent complete formulations and generalize across predictive scenarios.

We introduce the Composite Material Transformer (COMET), which encodes molecular structures, molar percentages and synthesis parameters in a transformer-based architecture. COMET is trained on the Lipid–RNA Nanoparticle Composition and Efficacy (LANCE) dataset of over 3,000 LNPs, including those with dual-ionizable lipids. COMET accurately predicted excluded samples and, via in silico screening of 50 million virtual LNPs, identified top candidates with high in vitro and in vivo expression. Unlike lipid-focused models, COMET showed versatility with polymeric materials, predicting efficacy from limited data. Using two smaller datasets (~10% of LANCE)—one in a gastrointestinal cell line, another post-lyophilization—we demonstrate COMET’s robust adaptability. With its flexibility and broad utility, COMET promises to accelerate complex drug product development.

## Results

### LNP design with COMET

**Fig. 1:**
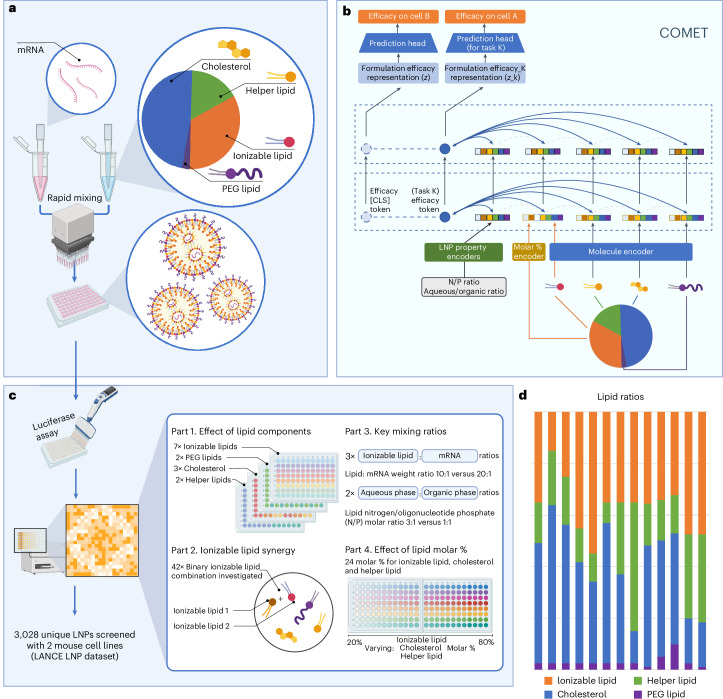
COMET predicts an LNP’s efficacy by inferring its formulation-wide properties, components’ molecular structures and compositions. **a**, LNPs are synthesized by mixing nucleic acid (for example, mRNA) with a lipid solution typically composed of four lipid classes. Key properties, such as efficacy, depend not only on the lipids’ structure but also on their relative ratios and other mixing parameters (for example, N/P and aqueous/organic ratio). **b**, COMET can predict properties of composite materials such as LNPs from their components, compositions and other parameters. **c**, With high-throughput screening, COMET’s training data are made up of four parts, each spanning a complementary LNP formulation space. **d**, Thirteen lipid molar ratios used in the majority of the LNP training dataset. Created with BioRender.com.

Composite materials such as LNPs comprise multiple components defined by the identity of constituent compounds, their relative ratios and formulation parameters such as nitrogen-to-phosphate (N/P) and mixing ratio (Fig. 1a). Previous studies often focused on a single component (for example, ionizable lipid)^25^; by contrast, we developed COMET to holistically represent LNPs and predict their efficacy via a flexible neural architecture (Fig. 1b). Lipid structures are encoded into molecular embeddings, while molar percentages are transformed into composition embeddings. These are concatenated to represent each lipid. Formulation-wide features, such as N/P and phase mixing ratios, are also embedded and fed into the model (Methods).

COMET adopts a transformer design similar to language models such as ChatGPT^30,31^, where chemical components and formulation features act as discrete tokens. It accommodates arbitrary numbers of components, including dual-ionizable lipid formulations. An LNP-level Classify ([CLS]) token attends to component and formulation vectors via self-attention^32^, with the final prediction made through a task-specific prediction head. For multitask learning, distinct CLS tokens and heads are used per task to capture differences across cell types while sharing model knowledge.

To train COMET, we use a pairwise ranking objective that learns to rank LNPs by efficacy (Methods). Noise augmentation improves robustness against experimental noise, and a label margin captures efficacy differences. In multitask settings, CAGrad helps align gradients across tasks^33^. We further enhance performance with an ensemble of COMET models, especially beneficial in low-data regimes^34,35^.

### LANCE dataset

To train COMET, we developed a high-throughput pipeline to generate the LANCE dataset. Each LNP in this dataset encapsulated a firefly luciferase (FLuc) messenger RNA (mRNA), and transfection efficacy was quantified by bioluminescence readouts. The LNPs were synthesized using automated fluid handling and tested in vitro. The LANCE dataset spans a wide design space structured in four parts: lipid identities (parts 1 and 2), synthesis parameters such as N/P and aqueous/organic mixing ratio (part 3), and lipid molar percentages (part 4) (Fig. 1c). Thirteen distinct molar ratios were used (Fig. 1d and Supplementary Table 13), generating over 6,000 labelled data points, including 3,028 LNPs evaluated in mouse DC2.4 and B16-F10 cells. Bioluminescence values were log-transformed and normalized between 0 and 1. Full methodological details are in Methods.

**Fig. 2:**
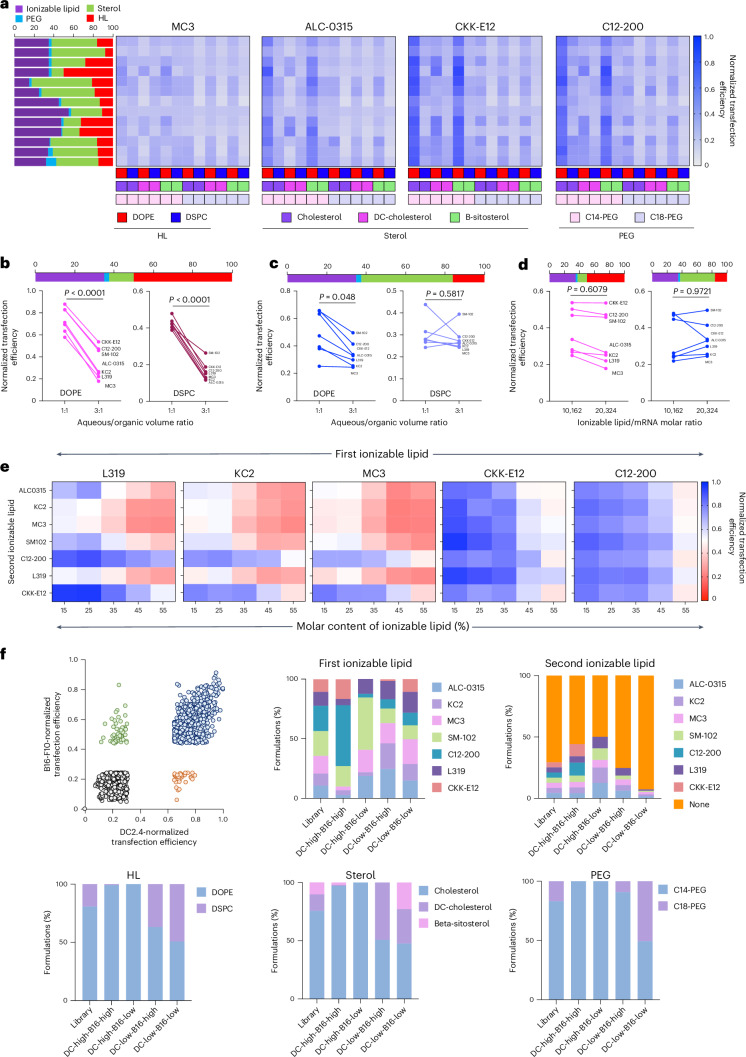
Effect of formulation parameters on LNP efficacy. **a**, Effect of lipid choice and ratio on LNP transfection efficacy in DC2.4 cells. HL, helper lipid. **b**,**c**, Effect of aqueous/organic volumetric ratio on the transfection efficacy of LNPs containing high percentage of helper lipid (**b**) and low percentage of helper lipid (**c**) in DC2.4 cells. **d**, Effect of ionizable lipid/mRNA weight ratios on transfection efficacy in DC2.4 cells. **e**, Effect of using two ionizable lipids on transfection efficacy in DC2.4 cells. **f**, Comparison of formulation performance in DC2.4 and B16-F10 cells. Data are derived from two biological and two technical replicates. Statistics in **b**–**d** were determined using unpaired two-tailed *t*-test.

In DC2.4 cells, LNPs with CKK-E12 or C12-200 as ionizable lipids outperformed those with DLin-MC3-DMA (Fig. 2a). Helper lipids (for example, 1,2-dioleoyl-sn-glycero-3-phosphoethanolamine (DOPE)), sterols (cholesterol/beta-sitosterol) and polyethylene glycol (PEG) lipids (C14-PEG) also had substantial effects on efficacy. Hence, the LANCE dataset successfully captured these previous observations^12,20,36,37^. Molar ratios had notable but formulation-specific effects on efficacy; no single ratio consistently outperformed across all lipid combinations. This highlights the need for context-specific optimization. Altering aqueous/organic phase ratios from 3:1 to 1:1 affected efficacy in helper-lipid-rich LNPs (Fig. 2b), although the effect diminished at lower helper lipid content (Fig. 2c) and was negligible for 1,2-distearoyl-sn-glycero-3-phosphocholine (DSPC)-based formulations. The N/P ratio, by contrast, showed no clear efficacy association (Fig. 2d). We further evaluated five-component formulations by adding a second ionizable lipid (3:2 ratio) alongside DOPE, cholesterol and C14-PEG. Potent ionizable lipids such as CKK-E12 and C12-200 enhanced weak lipids such as L319 or DLin-MC3-DMA (Fig. 2e). Notably, CKK-E12/L319 combinations outperformed both CKK-E12-only and dual-strong-lipid pairings, particularly at 25% total ionizable lipid content.

Cross-cell line comparison of DC2.4 and B16-F10 results revealed 772 formulations that were in the top 30th percentile in both (Fig. 2f), commonly containing C12-200, DOPE, cholesterol and C14-PEG. Formulations with selective activity were also identified: SM102 appeared frequently in DC2.4-high but B16-F10-low cases, while DC-cholesterol was enriched in the opposite group. In summary, transfection efficacy is governed not only by lipid identities but also by molar ratios and synthesis conditions—motivating the need for models such as COMET that can integrate and learn from multifactorial design spaces.

### Performance of COMET

**Fig. 3:**
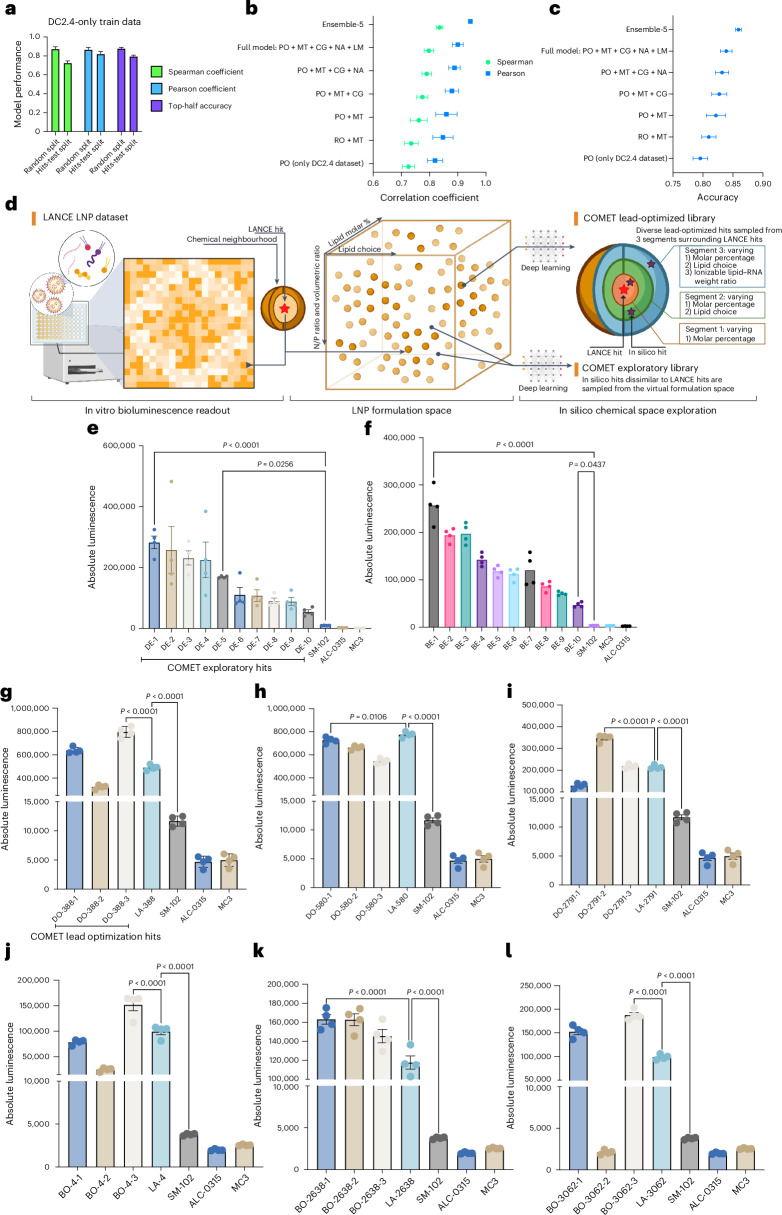
COMET predicts efficacy accurately and finds new hits. **a**, Performance of COMET on different DC2.4 test data splits after training on DC2.4 LNP efficacy data. **b**,**c**, Ablation results showing how different modules contribute to COMET’s ranking performance (**b**) and accuracy (**c**) on DC2.4 ‘hits-test’ test set. **d**, Schematic of in silico hit selection that begins with a large virtual LNP library, in silico screening with COMET and filtering based on LNPs’ properties such as efficacy and diversity. **e**,**f**, In vitro validation of exploratory in silico hits in DC2.4 (**e**) and B16-F10 (**f**) cells. **g**–**i**, Lead optimization around three top-performing LANCE LNPs in DC2.4 cells, namely LA-388 (**g**), LA-580 (**h**) and LA-2791 (**i**), was performed with COMET, each of which yielded three new formulations (denoted with the prefix DO) and evaluated experimentally. **j**–**l**, Lead optimization around three top-performing LNPs in B16-F10, namely LA-4 (**j**), LA-2638 (**k**) and LA-3062 (**l**), was performed with COMET, each of which yielded three new formulations (denoted with the prefix BO) and evaluated experimentally. Twenty replicates of training run with different random seeds were used for evaluation in **a**–**c**. Four technical replicates were used for **e**–**l**. Error bars are s.e.m. Statistical significances in **e**–**l** were determined using a one-way analysis of variance (ANOVA) with post-hoc Tukey test. MT, multitask; RO, regression objective; PO, pairwise ranking objective; CG, CAGrad; NA, noise augmentation; LM, label margin. Panel **d** created with BioRender.com.

We evaluated COMET on a random 20% test split of LNPs, with 10% used for validation and the remaining 70% for training. When trained to predict DC2.4 efficacy, COMET accurately ranked the test samples, achieving a Spearman coefficient of 0.873 and a Pearson coefficient of 0.866 (Fig. 3a). To simulate a more realistic drug discovery scenario, we curated a ‘hits-test’ split where the top 10% of DC2.4 LNPs were withheld as ‘hits’, alongside a random 10% of ‘non-hits’. COMET retained strong predictive power, yielding a Spearman coefficient of 0.725 and a Pearson coefficient of 0.820 (Fig. 3a). Its ability to classify ‘hits’ into the top half of ranked predictions reached 79.6% accuracy.

In a multitask learning set-up using both DC2.4 and B16-F10 labels, COMET’s performance improved on the DC2.4 test set, achieving a Spearman of 0.762 and a Pearson of 0.860 (pairwise ranking objective + multitask) model; Supplementary Fig. 1). These improvements scaled with the size of the additional B16-F10 data (Supplementary Fig. 2), highlighting the benefit of shared representation learning across related tasks^38^. We performed ablation studies to understand the contribution of each modelling component. Replacing the pairwise ranking objective with a regression objective slightly reduced performance (Fig. 3b,c). Model enhancements—ensemble learning, noise augmentation, label margin and CAGrad regularization—each contributed to performance gains, with ensembling having the greatest effect (Fig. 3b,c). Gains from ensembling plateaued beyond five models (Supplementary Fig. 3), and so we used an ensemble of five COMETs for all in silico screening.

To probe whether COMET learns meaningful structure–activity relationships, we performed adversarial perturbations. When lipid identities in training samples were partially shuffled, model performance degraded monotonically (Supplementary Fig. 4). More aggressive shuffling across lipid classes led to a further drop, and corrupting additional formulation parameters (for example, N/P ratio, molar % and phase ratios) impaired performance even more (Supplementary Fig. 5). COMET’s generalizability was tested by excluding LNPs containing selected ionizable lipids (MC3, SM-102 and CKK-E12) and a sterol (beta-sitosterol) from training. The model maintained good performance on this chemically distinct test set, with Pearson/Spearman correlations of 0.779/0.776 for DC2.4 and 0.502/0.509 for B16-F10 efficacy prediction (Supplementary Fig. 6a,b).

Lastly, we compared COMET against simpler baselines. Both random forest and COMET outperformed *k*-nearest neighbours, with COMET’s single-model performance comparable to random forest (Supplementary Fig. 7). An ensemble of COMETs offered improved correlation metrics for both cell lines, although top 50% classification accuracy remained similar to that of random forest.

To determine whether COMET learns transfection efficacy rather than proxying classic LNP properties, we analysed correlations between COMET predictions and nanoparticle characteristics such as encapsulation efficiency, size, polydispersity and zeta potential. COMET’s predictions were only weakly correlated with these properties—except for particle size (correlation 0.6530)—but were highly correlated with actual transfection data (>0.95; Supplementary Figs. 8 and 9). This confirms that COMET predicts efficacy itself, not just physical proxies.

### Experimental validation of COMET in silico hits

To evaluate COMET’s ability to discover effective formulations beyond LANCE, we screened a virtual library of nearly 50 million LNPs and validated selected in silico ‘hits’ experimentally (Fig. 3). Exploratory hits were chosen by excluding LNPs similar to top-performing LANCE formulations and then selecting chemically diverse candidates predicted by COMET to be highly efficacious (Methods, Fig. 3d and Supplementary Fig. 10).

All exploratory hits outperformed clinically approved LNPs^39^ (SM-102 (ref. ^15^), ALC-0315 (ref. ^16^) and DLin-MC3-DMA^40^) in both DC2.4 and B16-F10 cells (Fig. 3e,f). The top DC2.4 exploratory hit matched two of three top LANCE hits (Supplementary Fig. 11), while the best B16-F10 exploratory hit exceeded all three LANCE hits tested (Supplementary Fig. 12).

We next evaluated COMET’s ability to refine existing leads. Around selected LANCE hits, virtual candidates were generated by modifying lipid ratios, substituting components or changing N/P ratios (Methods and Fig. 3d). In DC2.4, COMET identified optimized formulations outperforming their parent in two of three cases (Fig. 3g–i); in B16-F10, all three optimized LNPs outperformed their respective parents (Fig. 3j–l).

### Adapting to new materials

**Fig. 4:**
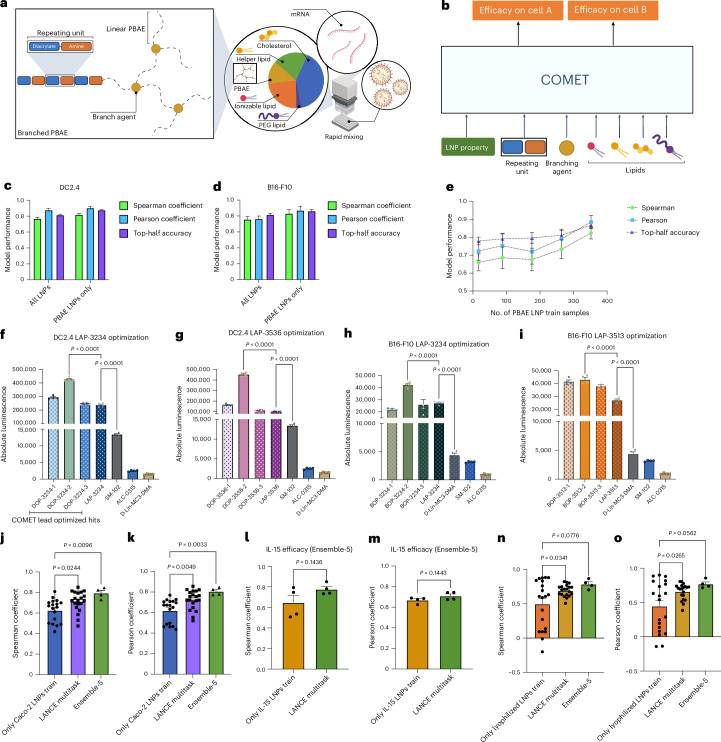
COMET can be adapted to applications of new material, a new cell type and stability. **a**, Structure of branched PBAEs. **b**, Incorporation of PBAE along with LNP features in COMET’s inference. **c**,**d**, Evaluation of COMET’s performance on PBAE LANCE test set and its subset containing only PBAE LNPs, in predicting DC2.4 efficacy (**c**) and B16-F10 efficacy (**d**). **e**, Effect of PBAE LNP training data size on predictive performance, averaged over both cell lines. **f**–**i**, In vitro validation of lead optimization in silico PBAE LNP candidates compared with the original LANCE PBAE hit and baseline LNPs for DC2.4 (**f**,**g**) and B16-F10 (**h**,**i**) cells. **j**,**k**, Evaluation of COMET’s performance on predicting LNPs’ efficacy in Caco-2, with Spearman (**j**) and Pearson (**k**) correlation. **l**,**m**, Evaluation of COMET’s performance on predicting LNPs’ efficacy in delivering IL-15 mRNA payload to HepG2, when evaluated as ensembles of five models, with Spearman (**l**) and Pearson (**m**) correlation. **n**,**o**, Evaluation of COMET’s performance on predicting degradation of LNPs’ efficacy after lyophilization, with Spearman (**n**) and Pearson (**o**) correlation. Twenty replicates were used for evaluation in **c**, **d** and **j**–**o**, except for Ensemble-5, which has four replicates. Error bars are s.e.m. Statistical significances in **f**–**i** were determined using a one-way ANOVA with post-hoc Tukey test. Statistical significances in **j**, **k**, **n** and **o** were determined using a one-way ANOVA with post-hoc Dunnett test. Statistics in **l** and **m** were determined using unpaired two-tailed *t*-test. Panels **a** and **b** created with BioRender.com.

To assess COMET’s adaptability beyond lipids, we extended it to branched poly(beta-amino esters) (PBAEs)^3,41^ (Fig. 4a), a class of polymers. A dataset of 454 polymer–LNPs (13 unique PBAEs) was added to LANCE. Each PBAE was represented by its diacrylate–amine unit and branching agent (Methods and Fig. 4b). In the ‘hits-test’ setting, COMET achieved Spearman coefficients of 0.767 (DC2.4) and 0.756 (B16-F10) (Fig. 4c,d). Notably, PBAE LNPs represented only 13% of the training data.

Even when trained on just 17 PBAE LNPs plus LANCE data, COMET achieved a mean Spearman of 0.660 across both cell types, improving to 0.824 with the full 352-sample PBAE set (Fig. 4e). We selected top-performing PBAE LNPs for further optimization using COMET (Methods). Optimized candidates showed higher efficacy than their parent formulations in both DC2.4 cases (Fig. 4f,g) and in one B16-F10 case (Fig. 4h,i).

### Adapting to new target cell and payload

To evaluate its capability to adapt to a new cell type, COMET was tested on a dataset of 295 LNPs screened in human Caco-2 cells. Activity poorly correlated with mouse cells (Supplementary Fig. 15), indicating a need for learning-based formulation strategies to quickly identify new formulations that are optimal for new settings. COMET trained on Caco-2 data achieved a Spearman coefficient of 0.639, which improved to 0.713 with LANCE multitask training, and to 0.794 (Pearson 0.806) using a five-model ensemble (Fig. 4j,k).

We further tested COMET on HepG2 cells transfected with interleukin (IL)-15 mRNA using 98 LNPs. With multitask ensemble training, we observed strong predictions (Pearson 0.709 and Spearman 0.775; Fig. 4l,m). Additional LANCE data did not improve single-model accuracy (Supplementary Fig. 16) but enhanced ensemble performance (Fig. 4l,m), likely owing to increased diversity among the COMET models when trained with additional LANCE data.

### Application in stabilization of LNPs

To address the instability of LNPs at ambient temperatures, we trained COMET to predict efficacy loss post-lyophilization (Methods). We synthesized 168 LNPs with variable lipids and 20% (w/v) sucrose as the stabilizer. Post-lyophilization data (Supplementary Fig. 17a) revealed that top performers included CKK-E12 and C12-200 as ionizable lipids, and DOPE as the helper lipid. Interestingly, these were not always top performers pre-lyophilization. DC-cholesterol-containing LNPs ranked higher post-lyophilization. Using 148 samples for training/validation and 20 for testing, COMET achieved a Spearman of 0.492. With LANCE multitask data, this increased to 0.705, and to 0.788 with five-model ensembles (Fig. 4n,o).

### In vivo screening of in silico hits

**Fig. 5:**
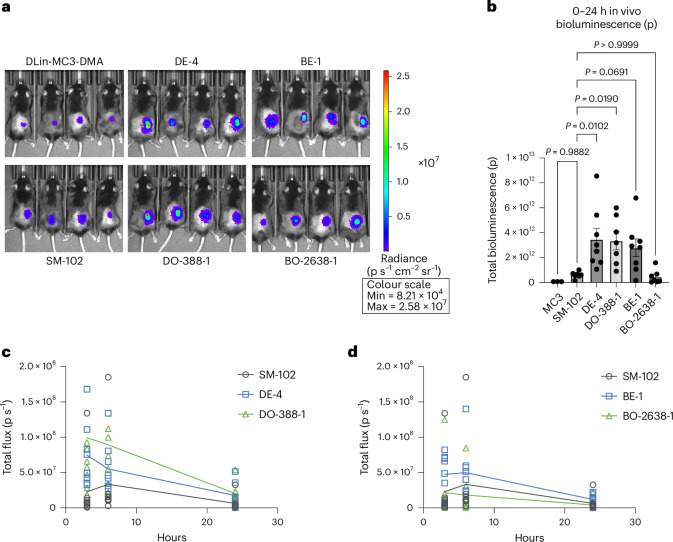
COMET-designed LNPs demonstrate in vivo efficacy. **a**, Bioluminescence image of clinical formulations and 4 COMET in silico hits 6 h after subcutaneous administration of 3 μg FLuc mRNA. **b**, Total bioluminescence, computed as area under the curve over 3 timepoints (3 h, 6 h and 24 h after subcutaneous administration of FLuc mRNA). **c**,**d**, Bioluminescence quantification of in silico hits that were optimized for efficacy in DC2.4 (**c**) and B16-F10 (**d**) and the SM-102 clinical baseline LNP at the same three timepoints. Four biological replicates were used for MC3 formulation, while seven to eight biological replicates were used to evaluate the other formulations in **b**. Error bars are s.e.m. Statistical significances in **b** were determined using a one-way ANOVA with post-hoc Dunnett test.

We selected one hit from each virtual LNP group (‘Experimental validation of COMET in silico hits’ section) for in vivo validation in mice. Compared with DLin-MC3-DMA and SM-102 clinical benchmarks, COMET hits yielded >40-fold and >5-fold higher bioluminescence, respectively (Fig. 5a,b), with faster transfection kinetics (Fig. 5c). Top-performing COMET hits (DE-4, DO-388-1 and BE-1) also had higher encapsulation efficiency (Supplementary Fig. 19a) and lower cytotoxicity than SM-102 (Supplementary Fig. 20).

In 1:1 in vivo comparisons with LANCE top hits, COMET-optimized LNPs matched DC2.4 benchmarks (Supplementary Fig. 18a,b), but underperformed slightly in B16-F10 (not significant), possibly owing to poor correlation between in vitro B16-F10 data and subcutaneous in vivo response (Supplementary Fig. 18c,d).

### Interpretation of COMET’s predictions

**Fig. 6:**
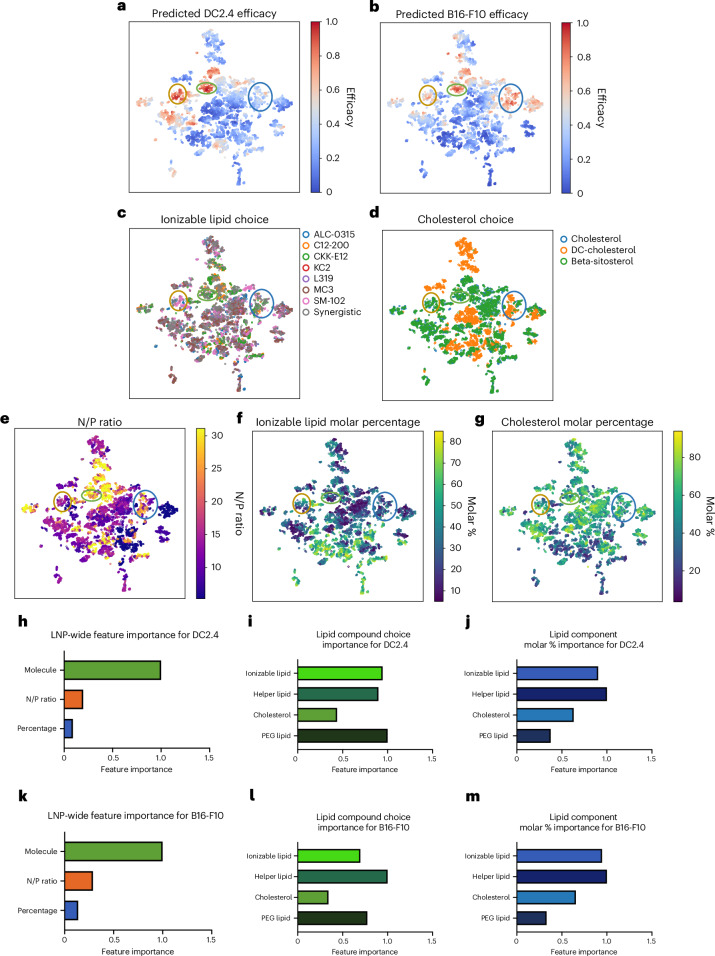
Interpreting COMET. **a**–**g**, t-SNE visualization of COMET representations of 10K virtual LNPs, each sample coloured by its predicted efficacy or feature value, that is, predicted DC2.4 (**a**) and B16-F10 (**b**) efficacy, ionizable lipid choice (**c**), cholesterol choice (**d**), N/P ratio (**e**), ionizable lipid molar percentage (**f**) and cholesterol molar percentage (**g**). **h**, COMET’s feature importance of LNP’s lipid molecule choice, N/P ratio and molar composition in the prediction of DC2.4 efficacy. **i**,**j**, Feature importance, broken down into lipid component types, for the choice of molecule (**i**) and molar percentage (**j**). **k**, COMET’s feature importance of LNP’s lipid molecule choice, N/P ratio and molar composition in the prediction of DC2.4 efficacy. **l**,**m**, Feature importance, broken down into lipid component types, for the choice of molecule (**l**) and molar percentage (**m**).

We used t-distributed stochastic neighbour embedding (t-SNE) to visualize how COMET encodes LNP compositional features to predict efficacy. As shown in Fig. 6a,b (for virtual LNPs) and Supplementary Fig. 22 (for LANCE LNPs), high-efficacy LNPs form distinct regional clusters within COMET. Notably, a green cluster contains LNPs efficacious in both DC2.4 and B16-F10, while yellow and blue clusters are specific to DC2.4 and B16-F10, respectively (Fig. 6a). Although ionizable lipid choices vary within each group, the DC2.4-specific cluster has a higher prevalence of SM-102 (Fig. 6c), and the B16-F10-specific cluster contains more LNPs with DC-cholesterol (Fig. 6d). The green cluster is enriched in high N/P ratio (25–30) formulations (Fig. 6e). Some of these patterns were observed in the LANCE data (Fig. 2), but others emerged only through COMET’s predictions.

In general, DOPE and C14-PEG are dominant among highly scored LNPs, suggesting that they are more optimal than DSPC and C18-PEG. Beta-sitosterol is overrepresented in high-scoring LNPs (Fig. 6d), supporting previous findings^36,42–44^. Two composition trends stood out: ionizable lipid % above 50% (Fig. 6f) and sterol % below 10% (Fig. 6g) were both detrimental to efficacy. Additional LNP feature visualizations are shown in Supplementary Fig. 21.

Using integrated gradients^45^, we identified features most influential to COMET’s predictions (Methods). For DC2.4, lipid identity was the most important factor, followed by N/P ratio and molar percentages (Fig. 6h). Among lipid classes, PEG lipid choice had the highest influence (Fig. 6i), indicating that switching from C18-PEG to C14-PEG improves efficacy predictions. Ionizable lipid choice was the next most critical, consistent with previous work^12,46–48^. For molar composition, ionizable and helper lipid percentages were most impactful (Fig. 6j). Similar trends held for B16-F10 (Fig. 6k–m), except that helper lipid choice ranked slightly higher than ionizable lipid choice.

We also analysed interactions between PBAE (Supplementary Section A.1) and synergistic ionizable lipid combinations (Supplementary Section A.2) with other formulation features. These interactions revealed that optimal material choices and molar percentages depend not only on the target cell type but also on the broader compositional context of the formulation.

## Conclusion

The design of COMET is motivated by the importance of not only the molecular structure of individual ingredients (for example, lipids) in drug products but also the interactions among compounds and their relative ratios. Its transformer-based architecture integrates multimodal features—including molecular structures, molar percentages and synthesis parameters—into a unified artificial intelligence framework. This enables COMET to learn LNP formulation features in a data-driven manner, without relying on manually selected physicochemical descriptors^27,28^. COMET accurately predicts LNP efficacy after training on LANCE, one of the largest LNP datasets so far^49–52^, and can distinguish top formulations from less efficacious ones.

While COMET consistently outperforms *k*-nearest neighbours, its advantage over random forest depends on dataset size and complexity. As larger, more diverse datasets emerge—especially with broader lipid chemistries—COMET’s deep learning architecture will likely offer increasing benefits. High-throughput methods are poised to accelerate this growth.

COMET’s flexible input format enables exploration of non-canonical formulations, such as dual-ionizable lipid LNPs or polymer–lipid hybrids (for example, branched PBAEs). It can screen massive virtual libraries to find formulations that differ substantially from known hits yet yield high performance—such as the L319-based BE-1 LNP. As LNP designs grow in complexity, COMET makes discovery more tractable.

In lead optimization, COMET identified stronger formulations in two out of three cases. In the one failure (LA-580), the parent formulation already had very high efficacy (Fig. 3h). This highlights that while COMET distinguishes top from mediocre LNPs well (‘Performance of COMET’ section), optimizing within a high-performing region requires even greater discriminative power. Adding more data from high-performing LNPs—especially through active learning—could improve this. COMET-predicted hits were validated across in vitro and in vivo settings. Since COMET is trained on in vitro data, and in vitro–in vivo correlation is known to be weak for LNPs^22,49^, not all predicted hits will succeed in vivo. Future integration with in vivo screening data^5,8^ may improve performance.

Beyond efficacy, COMET also predicts formulation stability post-lyophilization, despite limited data. This accuracy improves with multitask training using LANCE. Similar gains were observed in adapting COMET to new cell types (for example, Caco-2), underscoring the broad applicability of our approach. This is especially useful in contexts where assays are low throughput and datasets are small. The flexibility of COMET to handle multi-component inputs also allows for its extension beyond conventional LNPs. We demonstrated the model’s adaptability to formulations with non-lipid materials (for example, branched PBAEs) and its utility across multiple cell types. COMET’s architecture may also support links to other areas of nanotechnology where multi-component formulations are critical, such as co-delivery of multiple cargos, immunomodulatory nanoparticle design or materials for tissue engineering. In such contexts, COMET’s compositional encoding and multitask learning structure could be adapted to jointly predict multiple endpoints, including efficacy, toxicity or stability.

Coupled with advances in high-throughput science, we hope that COMET will become an essential tool for formulation development and discovery of knowledge in this field.

## Methods

### COMET details

This section describes the model architecture and training algorithms of COMET. Pseudocode for inference is provided in Algorithm S1.

#### COMET model architecture

Lipid molecular structures are encoded into high-dimensional vectors (molecular embeddings), while scalar compositional features are encoded using a Gaussian-based encoder^53^. Continuous formulation-wide parameters (for example, N/P ratio and volumetric mix ratio) are encoded with Gaussian layers; categorical inputs use one-hot embeddings.

The transformer uses a [CLS] token to aggregate input features across multiple attention layers. For multitask learning, each cell type is assigned a separate [CLS] token and prediction head, enabling task-specific outputs while sharing LNP-level representation learning.

##### Molecular encoder

COMET is compatible with various molecular encoders; here we use Uni-Mol^11^, pretrained to recover masked atom types and corrupted three-dimensional coordinates. It offers strong property prediction performance and is used with default hyperparameters (from https://github.com/dptech-corp/Uni-Mol/tree/main/unimol). Pretrained weights are frozen during COMET training. Each compound is encoded into a 512-dimensional vector using atom types and coordinates.

Lipid molar percentages are encoded into 128-dimensional vectors using a shared Gaussian layer. Each component is further assigned a 128-dimensional one-hot embedding (${z}_{k}^{{\rm{type}}}$) to distinguish lipid classes. These are concatenated and projected through a two-layer MLP into a 256-dimensional component representation.

##### N/P ratio and volumetric ratio

N/P ratio is encoded using a separate 256-dimensional Gaussian layer (*z*_N/P_). Aqueous/organic ratios, treated as categorical variables, are one-hot encoded (*z*_phase_) with 256 dimensions.

##### CLS token and prediction head

Each cell type uses a learned [CLS] token (*z*_CLS_) of dimension 256. These aggregate component and formulation-wide token representations across *N*_block_ transformer layers via attention^54^. Final predictions are made by passing the [CLS] token through a two-layer MLP (MLP_predict_).

##### Transformer blocks

Each block follows a Pre-LayerNorm structure^55^ composed of layernorm → self-attention → MLP with residual connections.

#### Training details

The model is trained with a binary ranking objective^56^ where, given a pair of LNP samples, the model learns to predict a larger efficacy score for the LNP that has a higher efficacy label value from the other LNP:

1 $${{\mathcal{L}}}_{\mathrm{ranking}}=-\log \left(\sigma (\;{f}_{\theta }({x}_{\mathrm{h}})-{f}_{\theta }({x}_{\mathrm{l}}))\right.$$

where *x*_h_ and *x*_l_ are high- and low-efficacy LNPs and *f*_*θ*_ is COMET’s scoring function. Training uses a batch size of 64 (2,016 pairwise comparisons per batch).

##### Conflict-averse gradient descent

Conflict-averse gradient descent (CAGrad)^33^ mitigates conflicting gradients in multitask settings. We apply CAGrad with a coefficient of 0.2 to stabilize training across tasks.

##### Noise augmentation

To address noise in the experimental data, especially in the fluid handling process, we augment the molar percentage with Gaussian noise proportionate to its value where the standard deviation of the noise is 10% of actual molar percentage.

##### Label margin

From the label values, we can tell not only which LNP is better than another but also by how much. To train the model to learn this additional knowledge, we include a margin term^57^ in the binary ranking objective:

2 $${{\mathcal{L}}}_{\mathrm{ranking}}=-\log \left({\rm{Sigmoid}}(\;{f}_{\theta }({x}_{\mathrm{h}})-{f}_{\theta }({x}_{\mathrm{l}})-{\lambda }_{\mathrm{margin}}(\;{y}_{\mathrm{h}}-{y}_{\mathrm{l}}))\right.$$

where *y*_h_ and *y*_l_ are the (efficacy) label values of the more efficacious and less efficacious LNP, respectively, and *λ*_margin_ controls how much this objective dominates the training. We use *λ*_margin_ = 0.01 in our experiments.

##### Ensembling

For in silico evaluation (Fig. 3e–l), the ensemble is formed by *N*_model_ models trained with the same hyperparameters and dataset (train/valid/test split) but weights initialized with different random seeds. For the ensemble deployed to infer virtual LNPs, 5 different train (80%)/valid (20%) splits are made in a fivefold manner and each model in the ensemble is trained on a different fold. To ensure that ensembled scores are not biased towards models with high variance, the predicted scores from each model are normalized by making their scores for the LANCE LNPs fit a normal distribution with mean 0 and standard deviation 1 before ensembling. More specifically, for each model, this is done by inferring the predicted scores on all the LANCE LNPs and using the mean (mean_*i*_) and standard deviation (std_*i*_) of LANCE LNPs’ scores to compute the normalized scores ${y}_{i}^{{\prime} \mathrm{normalized}}$ through

3 $${y}_{i}^{{\prime} \mathrm{normalized}}=\frac{{y}_{i}^{{\prime} }-{\mathrm{mean}}_{i}}{\mathrm{std}_{i}},\quad i \sim \{1,{.}{.}{.},{N}_{{\rm{model}}}\}$$

The final ensemble score is the mean of all models’ normalized scores:

4 $${y}^{{\prime} \mathrm{ensemble}}=\frac{1}{{N}_{{\rm{model}}}}\mathop{\sum }\limits_{i}^{{N}_{{\rm{model}}}}{y}_{i}^{{\prime} \mathrm{normalized}}$$

COMET is implemented in PyTorch and trained with NVIDIA V100 GPUs.

#### *k*-Nearest neighbours and random forest model details

The *k*-nearest neighbours and random forest models are implemented with the scikit-learn (https://scikit-learn.org/) package, with default hyperparameters. More specifically, the *k*-nearest neighbours model uses *n* = 5 nearest neighbours while the random forest model uses *n* = 100 estimators (trees).

### LANCE dataset details

LANCE comprises four parts spanning orthogonal LNP design dimensions: lipid component identities, molar percentages, synthesis parameters (for example, N/P and aqueous/organic volumetric ratios) and high-resolution molar sweeps.

Seven ionizable lipids, three sterols, two helper lipids and two PEG lipids were used (Supplementary Table 14), reflecting the focus of current research^12,42^. To study molar % effects, we designed 13 lipid ratios by varying one lipid class at a time from a reference BASE ratio (Fig. 1d), based on ref. ^20^. For instance, ratios I1–I4 modify ionizable lipid %, C1–C3 adjust cholesterol (compensated by helper lipid), and P1–P3 alter PEG lipid %, while the remaining modify multiple components (Supplementary Table 13).

#### Part 1 (lipid choice)

To examine lipid identity effects, we generated 84 combinations from all permutations of 7 ionizable lipids, 3 sterols, 2 helper lipids and 2 PEG lipids. Paired with 13 molar ratios, this results in 1,092 possible LNPs; 1,066 were tested. After removing 91 overlapping with part 2, this part yielded 975 unique LNPs.

#### Part 2 (ionizable lipid synergy)

Following studies suggesting synergy from dual-ionizable lipid formulations^58^, we created LNPs with 60:40 molar splits across all ionizable lipid pairs, distributed across 13 lipid ratios. This yielded 637 additional LNPs.

#### Part 3 (key synthesis parameters)

To explore synthesis effects, we introduced variation in ionizable lipid/RNA weight ratios (10:1, 15:1 and 20:1) and aqueous/organic phase ratios (1:1 and 3:1). Weight ratios were adjusted by molar mass to maintain equivalent molar %. These parameters were later converted to N/P ratios for model input. This part includes 924 LNPs.

#### Part 4 (molar percentage sweeps)

To study finer-grained molar % effects, we created 24 evenly spaced intervals from 10% to 80% for ionizable lipid, cholesterol and helper lipid, generating 492 LNPs across 3 focused sweeps.

#### Formulation ratios

Single-ionizable LNPs span 18 unique N/P ratios, derived from 3 ionizable lipid/RNA weight ratios and 7 ionizable lipids. Dual-ionizable formulations add 63 more, totalling 81 N/P ratios. In molar terms, 13 base lipid ratios and 72 sweep ratios (24 per lipid class) result in 85 total molar compositions.

#### LNP synthesis

LNPs were synthesized by mixing lipid–ethanol and mRNA–citrate buffer phases, incubated at 4 °C for 10 min. Automated handling was performed on the Tecan Fluent platform. For animal studies, LNPs were mixed, incubated on ice for 10 min and dialysed overnight at 4 °C in PBS (Slide-A-Lyzer, ThermoFisher).

#### Materials

FLuc mRNA (L-7202, Trilink); lipids (Cayman Chemicals, Avanti); luciferase assay (Steady-Glo, E2550) and Agilent BioTek plate reader for readout. alamarBlue was used for viability assays.

#### Data processing

Each 96-well plate included a ‘standard’ LNP. Raw luminescence values were normalized to the standard and averaged across four replicates (two biological, two technical). Mean values were log-transformed and min–max normalized to [0, 1].

We have represented several key features of the LANCE dataset in Fig. 2. Below, we explain how these key features were extracted from LANCE. For Fig. 2a, part 1 formulations were selected. For the four ionizable lipids (ALC-0315, DLin-MC3-DMA, C12-200 and CKK-E12), we had 156 formulations containing 2 helper lipids, 3 sterol lipids and 2 PEG lipids (that is, 2 × 2 × 3 = 12 combinations) at 13 molar ratios (12 × 13 = 156 formulations).

For Fig. 2b,c, part 3 formulations containing one ionizable lipid, cholesterol and C14-PEG were selected. Two molar ratios of the lipid components (which are shown in the figure) were studied. The ionizable lipid to mRNA molar ratio was 10,162. The aqueous to organic volume ratio was varied. For Fig. 2d, part 3 formulations containing one ionizable lipid, DOPE, cholesterol and C14-PEG were selected. Two molar ratios of the lipid components (which are shown in the figure) were studied. The organic to aqueous volume ratio was held at 1:3.

Figure 2e was generated from part 2 data. Only formulations containing DOPE, cholesterol and C14-PEG were used for the graph. The name of the first ionizable lipid was listed as the title of graph and the second ionizable lipid name was the row name. The total molar content of the ionizable lipids was the column name. The molar ratio of ionizable lipid 1/ionizable lipid 2 is 1.5. The molar ratio of DOPE/cholesterol was 0.34. The molar % of C14-PEG was 2.5%. The molar ratio of ionizable lipid/mRNA was 10,162. The entire library was used to construct Fig. 2f. We calculated the normalized transfection efficacy for the 30th and 70th percentile formulations in B16-F10 and DC2.4 cells. These values were as follows: 70th percentile, B16-F10 = 0.43887; 30th percentile, B16-F10 = 0.24315; 70th percentile DC2.4 = 0.64623; 30th percentile DC2.4 = 0.30946. Formulations above and below these values in the respective cell lines were selected and are plotted in Fig. 2f.

### In vitro validation details

The LNPs are named according to the groups to which they belong. A summary of the prefixes used here is given in Supplementary Table 16.

#### Clinically approved LNP baselines

The recipes for the 3 clinical LNP baselines are based on the literature^39^ and synthesized in an aqueous/organic volumetric of 3:1 following what is typically used in previous work.

#### Top LANCE LNP hits baselines

To find strong and reliable LNP baselines from LANCE, we randomly select 10 LNP formulations from the 90th percentile for each cell line to again screen them with the respective cell line to check for reproducibility. Among these ten formulations, three LNPs with their normalized efficacy value closest to their original LANCE efficacy label values were selected as LANCE baseline LNPs.

#### Exploratory LNP library

To span a vast formulation space, the virtual library was generated by enumerating through possible LNP features such as lipid choices, their molar percentages and key synthesis parameters such as N/P ratios and aqueous/organic volumetric ratios, according to Supplementary Table 15. To find LNPs that are different from the hits in the LANCE dataset, formulations within a 10% L1 distance lipid molar percentage neighbourhood of any top 10% most efficacious LANCE hits were excluded. After this step, the exploratory library has 27,354,600 and 34,539,960 formulations for DC2.4 and B16-F10, respectively. An ensemble of five COMET models predicted efficacy in both cell lines. The top 0.1% highest-scoring LNPs were selected (34,529 B16-F10 and 27,354 DC2.4).

The next step removes formulations based on uncertainty in COMET prediction. We capture the level of uncertainty by first computing the standard deviation (*σ*) between the models’ prediction (${y}_{i}^{{\prime} \mathrm{normalized}}$ in equation (3)) within the ensemble. We then scale the standard deviation by division with a non-negative predicted efficacy term to get a relative uncertainty value (*u*_rel_):

5 $${u}_{{\rm{rel}}}=\frac{\sigma }{{\hat{y}}^{\rm{ensemble}}},\quad {\hat{y}}^{\rm{ensemble}}={y}^{{\prime} \rm{ensemble}}-{y}^{{\prime} \rm{ensemble,min,LANCE}}$$

where ${y}^{{\prime} \mathrm{ensemble,min,LANCE}}$ is the minimum ensemble score among the LANCE LNPs. Any formulations with negative ${\hat{y}}^{\mathrm{ensemble}}$ term were dropped. Supplementary Fig. 13 shows the distribution of this relative uncertainty value. Formulations with largest 50% relative uncertainty values were removed, leaving 17,269 B16-F10 and 13,677 DC2.4 formulations.

To promote chemical diversity, *K*-means clustering (on 14-dimensional vectors encoding lipid molar percentages) grouped these candidates into 10 clusters. Clustering was repeated 1,000 times to stabilize assignments. The highest-scoring formulation in each cluster was selected, resulting in ten diverse in silico hits per cell line (Supplementary Tables 17 and 18).

#### Lead optimization LNP library

For each cell type, three top LANCE hits (from ‘Top LANCE LNP hits baselines’ section) were used as starting points. Around each, virtual candidates were generated by (1) exploring within a 20% L1 molar percentage distance, (2) substituting at least one lipid (6 ionizable lipids, 2 cholesterols, 1 helper and 1 PEG) and (3) altering the N/P ratio.

To generate three diverse candidates per lead, we segmented the neighbourhood into three zones: (1) molar % segment (within 20% L1, no lipid changes), (2) substitute-lipid segment (within 20% L1, but with at least one different lipid) and (3) N/P ratio segment (differing N/P ratio). From each zone, the top predicted LNP was selected (Fig. 3d, right). This yielded three optimized LNPs per lead. The virtual library size ranged from 1.5 million (single-ionizable lipid) to 9 million (dual-ionizable lipid) candidates. The sixfold increase in dual-ionizable lipid cases arises from combinatorial enumeration: each minor ionizable lipid was paired with six major ones. By contrast, single-ionizable lipid compositions require no pairing. The final selected formulations for validation are listed in Supplementary Tables 19 and 20.

### PBAE synthesis

The compositions and molar ratios of amines, diacrylates and branching agents are listed in Supplementary Table 21. To synthesize PBAE polymers, the combination of the amines, diacrylates and branching agents were used. In brief, in a 20 ml glass vial, the entire weight of diacrylate and branching agent was added. Then, the solvent (dimethylformamide) was added to the reaction mixture. Later, the reaction vials were placed on a hotplate at 90 °C. After 24 h, the vials were removed from the hotplate and cooled to room temperature. The amines were added to the reaction vial and placed back on the hotplate at 90 °C and the reaction was allowed to proceed for 48 h. Finally, the vials were removed from the hotplate and allowed to cool to room temperature. Then, the reaction mixture was added (drop-by-drop) into a beaker containing ~150 ml ice-cold diethyl ether (~10× excess volume). The collected samples were transferred to 50 ml tubes and centrifuged at 1,000 × *g* for 3 min to pellet the polymer. Later, the supernatant was removed and dissolved in the minimal possible volume of dimethylformamide. This purification step was repeated three times. Final polymers were dried under vacuum and solubility tested in ethanol.

### Representing PBAEs in COMET

PBAEs were represented as a combination of their diacrylate–amine repeating unit and branching agent, each with unique component-type embeddings. The repeating unit was treated as a fifth molar component type alongside lipids, with its molar concentration estimated from polymer weight and molecular weight. Total molar percentages of PBAE and lipids sum to 100%. Inference proceeds as in lipid-only LNPs (‘COMET details’ section).

### COMET PBAE LNP lead optimization hits

Two top-performing PBAE LNPs per cell type were used as starting points. Around each, virtual candidates were generated by (1) exploring within a 20% L1 molar percentage neighbourhood, (2) substituting lipids (6 ionizable lipids, 2 sterols, 1 helper and 1 PEG) and (3) converting to dual-ionizable compositions. To select three diverse candidates, we defined three non-overlapping segments: one within the 20% L1 distance but must have the same lipid choices, one with at least one different lipid compound and one with a dual-ionizable lipid configuration. The top predicted LNP from each segment was chosen (Fig. 3d, right). Final hits are detailed in Supplementary Tables 22 and 23.

### Human IL-15 screening

The IL-15 mRNA is synthesized via in vitro transcription with a HiScribe T7 mRNA kit with CleanCap Reagent AG (E2080S) from New England Biolabs, with 5-methoxy-UTP (N-1093) from Trilink. The LNP transfection is done at an mRNA concentration of 0.25 µg ml^−1^ in the 96-well plate format. The Human IL-15 expression level is measured with Human IL-15 Uncoated ELISA (88-7620) procured from Invitrogen, after 16 h of incubation of HepG2 cells with LNP. Raw efficacy data are normalized, similar to bioluminescence data mentioned above, before used as dataset for machine learning experiments. This dataset (20%) is randomly split into test set, while the rest is used as the train and validation sets.

### Lyophilization of LNPs

The LNPs are synthesized in a tris buffer (5 mM tris buffer, pH 8). After synthesis, the LNP formulations are frozen at −80 °C for 2 h before undergoing the following lyophilization process: equilibrate at −40 °C for 2 h, in atmosphere → −40 °C for 21 h, in vacuum → 25 °C for 2 h, in vacuum. Labconco FreeZone 6 l with a Stoppering Tray Dryer was used for lyophilization.

### Degradation in the efficacy

Post-lyophilization efficacy values were computed and normalized similarly to the LANCE label values (‘Data processing’ section). The degradation of efficacy owing to lyophilization was calculated by subtracting the post-lyophilization efficacy values score from the LANCE B16-F10 values.

### Animal experiments

Animal experiments for this study were approved by the Massachusetts Institute of Technology Institutional Animal Care and Use Committee and were consistent with local, state and federal regulations as applicable. Female C57BL/6J mice (000664, The Jackson Laboratory) were used in the experiments. For imagining, d-luciferin (LUCK-1G, Gold Biotechnology) solubilized in PBS was administered via intraperitoneal injection and the mice were imaged using an IVIS imaging system (PerkinElmer).

### t-SNE visualization

We selected the COMET model most correlated (Spearman) with ensemble scores across a random virtual LNP subset. LNP features for t-SNE were the final [CLS] token representations. To ensure even distribution across ionizable types, dual-ionizable lipid LNPs were treated as a distinct class, and 1,250 LNPs per class (8 total) were randomly sampled (10,000 total).

### Integrated gradients implementation

To execute integrated gradients (IG) with COMET’s multimodal inputs, we adapted the Captum library. IG computes attribution by integrating gradients along a path from reference to input. Feature attributions were computed per LNP, baseline-subtracted and averaged across each group. Non-PBAE LANCE LNPs were used as the baseline. Attribution scores were normalized (max = 1) and averaged across ensemble models.

### Reporting summary

Further information on research design is available in the Nature Portfolio Reporting Summary linked to this article.

## Online content

Any methods, additional references, Nature Portfolio reporting summaries, source data, extended data, supplementary information, acknowledgements, peer review information; details of author contributions and competing interests; and statements of data and code availability are available at 10.1038/s41565-025-01975-4.

## Supplementary information

Supplementary Information Supplementary Figs. 1–22, discussion and Tables 1–23.

Reporting Summary

## Data Availability

The data supporting the findings of this study are available within the paper and its Supplementary Information files. Should any raw data files be needed in another format, they are available from the corresponding authors upon reasonable request.

## Code availability

Source code for the COMET model, trained weights and inference scripts are available at https://github.com/alvinchangw/COMET.

## References

[1] Cheng, Q. et al. Selective organ targeting (SORT) nanoparticles for tissue-specific mRNA delivery and CRISPR–Cas gene editing. *Nat. Nanotechnol.* **15**, 313–320 (2020). 32251383 10.1038/s41565-020-0669-6 PMC7735425

[2] Ball, R. L., Bajaj, P. & Whitehead, K. A. Achieving long-term stability of lipid nanoparticles: examining the effect of pH, temperature, and lyophilization. *Int. J. Nanomed.* **12**, 305–315 (2016). 10.2147/IJN.S123062 PMC5221800 28115848

[3] Abramson, A. et al. Oral mRNA delivery using capsule-mediated gastrointestinal tissue injections. *Matter* **5**, 975–987 (2022).

[4] Reker, D. et al. ‘Inactive’ ingredients in oral medications. *Sci. Transl. Med.* **11**, 6753 (2019). 10.1126/scitranslmed.aau6753 PMC7122736 30867323

[5] Dahlman, J. E. et al. Barcoded nanoparticles for high throughput in vivo discovery of targeted therapeutics. *Proc. Natl Acad. Sci. USA* **114**, 2060–2065 (2017). 28167778 10.1073/pnas.1620874114 PMC5338412

[6] Sago, C. D. et al. High-throughput in vivo screen of functional mRNA delivery identifies nanoparticles for endothelial cell gene editing. *Proc. Natl Acad. Sci. USA*10.1073/pnas.1811276115 (2018). 10.1073/pnas.1811276115 PMC6196543 30275336

[7] Guimaraes, P. P. G. et al. Ionizable lipid nanoparticles encapsulating barcoded mRNA for accelerated in vivo delivery screening. *J. Control. Release* **316**, 404–417 (2019). 31678653 10.1016/j.jconrel.2019.10.028 PMC7032071

[8] Rhym, L. H., Manan, R. S., Koller, A., Stephanie, G. & Anderson, D. G. Peptide-encoding mRNA barcodes for the high-throughput in vivo screening of libraries of lipid nanoparticles for mRNA delivery. *Nat. Biomed. Eng.* **7**, 901–910 (2023). 37127709 10.1038/s41551-023-01030-4

[9] Yang, K. et al. Analyzing learned molecular representations for property prediction. *J. Chem. Inf. Model.* **59**, 3370–3388 (2019). 31361484 10.1021/acs.jcim.9b00237 PMC6727618

[10] Ross, J. et al. Large-scale chemical language representations capture molecular structure and properties. *Nat. Mach. Intell.* **4**, 1256–1264 (2022).

[11] Zhou, G. et al. Uni-Mol: a universal 3D molecular representation learning framework. In *The Eleventh International Conference on Learning Representations* (2023).

[12] Hou, X., Zaks, T., Langer, R. & Dong, Y. Lipid nanoparticles for mRNA delivery. *Nat. Rev. Mater.* **6**, 1078–1094 (2021). 34394960 10.1038/s41578-021-00358-0 PMC8353930

[13] Han, X. et al. An ionizable lipid toolbox for RNA delivery. *Nat. Commun.* **12**, 7233 (2021). 34903741 10.1038/s41467-021-27493-0 PMC8668901

[14] Mitchell, M. J. et al. Engineering precision nanoparticles for drug delivery. *Nat. Rev. Drug Discov.* **20**, 101–124 (2021). 33277608 10.1038/s41573-020-0090-8 PMC7717100

[15] Baden, L. R. et al. Efficacy and safety of the mRNA-1273 SARS-CoV-2 vaccine. *N. Engl. J. Med.* **384**, 403–416 (2021). 33378609 10.1056/NEJMoa2035389 PMC7787219

[16] Polack, F. P. et al. Safety and efficacy of the BNT162b2 mRNA Covid-19 vaccine. *N. Engl. J. Med.* **383**, 2603–2615 (2020). 33301246 10.1056/NEJMoa2034577 PMC7745181

[17] Swingle, K. L., Hamilton, A. G. & Mitchell, M. J. Lipid nanoparticle-mediated delivery of mRNA therapeutics and vaccines. *Trends Mol. Med.* **27**, 616–617 (2021). 33836968 10.1016/j.molmed.2021.03.003

[18] Chaudhary, N., Weissman, D. & Whitehead, K. A. mRNA vaccines for infectious diseases: principles, delivery and clinical translation. *Nat. Rev. Drug Discov.* **20**, 817–838 (2021). 34433919 10.1038/s41573-021-00283-5 PMC8386155

[19] Tenchov, R., Bird, R., Curtze, A. E. & Zhou, Q. Lipid nanoparticles—from liposomes to mRNA vaccine delivery, a landscape of research diversity and advancement. *ACS Nano* **15**, 16982–17015 (2021). 34181394 10.1021/acsnano.1c04996

[20] Kauffman, K. J. et al. Optimization of lipid nanoparticle formulations for mRNA delivery in vivo with fractional factorial and definitive screening designs. *Nano Lett.* **15**, 7300–7306 (2015). 26469188 10.1021/acs.nanolett.5b02497

[21] Hassett, K. J. et al. Optimization of lipid nanoparticles for intramuscular administration of mRNA vaccines. *Mol. Ther. Nucleic Acids* **15**, 1–11 (2019). 30785039 10.1016/j.omtn.2019.01.013 PMC6383180

[22] Lokugamage, M. P. et al. Optimization of lipid nanoparticles for the delivery of nebulized therapeutic mRNA to the lungs. *Nat. Biomed. Eng.* **5**, 1059–1068 (2021). 34616046 10.1038/s41551-021-00786-x PMC10197923

[23] Melamed, J. R. et al. Ionizable lipid nanoparticles deliver mRNA to pancreatic β cells via macrophage-mediated gene transfer. *Sci. Adv.* **9**, 1444 (2023). 10.1126/sciadv.ade1444 PMC9882987 36706177

[24] Reker, D. et al. Computationally guided high-throughput design of self-assembling drug nanoparticles. *Nat. Nanotechnol.* **16**, 725–733 (2021). 33767382 10.1038/s41565-021-00870-y PMC8197729

[25] Xu, Y. et al. AGILE platform: a deep learning powered approach to accelerate LNP development for mRNA delivery. *Nat. Commun.* **15**, 6305 (2024). 39060305 10.1038/s41467-024-50619-z PMC11282250

[26] Li, B. et al. Accelerating ionizable lipid discovery for mRNA delivery using machine learning and combinatorial chemistry. *Nat. Mater.*10.1038/s41563-024-01867-3 (2024). 10.1038/s41563-024-01867-3 38740955

[27] Cheng, L. et al. Machine learning elucidates design features of plasmid deoxyribonucleic acid lipid nanoparticles for cell type-preferential transfection. *ACS Nano* **18**, 28735–28747 (2024). 39375194 10.1021/acsnano.4c07615 PMC11512640

[28] Ding, D. Y., Zhang, Y., Jia, Y. & Sun, J. Machine learning-guided lipid nanoparticle design for mRNA delivery. In *2023 ICML Workshop on Computational Biology* (2023).

[29] Lewis, M. M., Beck, T. J. & Ghosh, D. Applying machine learning to identify ionizable lipids for nanoparticle-mediated delivery of mRNA. Preprint at *bioRxiv*10.1101/2023.11.09.565872 (2023).

[30] Achiam, J. et al. GPT-4 technical report. Preprint at https://arxiv.org/abs/2303.08774 (2023).

[31] Gemini Team Google et al. Gemini: a family of highly capable multimodal models. Preprint at https://arxiv.org/abs/2312.11805 (2023).

[32] Vaswani, A. et al. Attention is all you need. In *Proc. 31st International Conference on Neural Information Processing Systems* 6000–6010 (2017).

[33] Liu, B., Liu, X., Jin, X., Stone, P. & Liu, Q. Conflict-averse gradient descent for multi-task learning. *Adv. Neural Inf. Process. Syst.* **34**, 18878–18890 (2021).

[34] Dietterich, T. G. Ensemble methods in machine learning. In *International Workshop on Multiple Classifier Systems* 1–15 (Springer, 2000).

[35] Ganaie, M. A., Hu, M., Malik, A., Tanveer, M. & Suganthan, P. Ensemble deep learning: a review. *Eng. Appl. Artif. Intell.* **115**, 105151 (2022).

[36] Patel, S. et al. Naturally-occurring cholesterol analogues in lipid nanoparticles induce polymorphic shape and enhance intracellular delivery of mRNA. *Nat. Commun.* **11**, 983 (2020). 32080183 10.1038/s41467-020-14527-2 PMC7033178

[37] Zhu, X. et al. Surface de-PEGylation controls nanoparticle-mediated siRNA delivery in vitro and in vivo. *Theranostics* **7**, 1990 (2017). 28638484 10.7150/thno.18136 PMC5479285

[38] Zhang, Y. & Yang, Q. A survey on multi-task learning. *IEEE Trans. Knowl. Data Eng.* **34**, 5586–5609 (2021).

[39] Schoenmaker, L. et al. mRNA-lipid nanoparticle COVID-19 vaccines: structure and stability. *Int. J. Pharm.* **601**, 120586 (2021). 33839230 10.1016/j.ijpharm.2021.120586 PMC8032477

[40] Adams, D. et al. Patisiran, an RNAi therapeutic, for hereditary transthyretin amyloidosis. *N. Engl. J. Med.* **379**, 11–21 (2018). 29972753 10.1056/NEJMoa1716153

[41] Akinc, A., Anderson, D. G., Lynn, D. M. & Langer, R. Synthesis of poly(β-amino ester)s optimized for highly effective gene delivery. *Bioconjug. Chem.* **14**, 979–988 (2003). 13129402 10.1021/bc034067y

[42] Eygeris, Y., Patel, S., Jozic, A. & Sahay, G. Deconvoluting lipid nanoparticle structure for messenger RNA delivery. *Nano Lett.* **20**, 4543–4549 (2020). 32375002 10.1021/acs.nanolett.0c01386

[43] Medjmedj, A. et al. In cellulo and in vivo comparison of cholesterol, beta-sitosterol and dioleylphosphatidylethanolamine for lipid nanoparticle formulation of mRNA. *Nanomaterials* **12**, 2446 (2022). 35889670 10.3390/nano12142446 PMC9317807

[44] Douka, S. et al. Lipid nanoparticle-mediated messenger RNA delivery for ex vivo engineering of natural killer cells. *J. Control. Release* **361**, 455–469 (2023). 37567506 10.1016/j.jconrel.2023.08.014

[45] Sundararajan, M., Taly, A. & Yan, Q. Axiomatic attribution for deep networks. In *International Conference on Machine Learning* 3319–3328 (PMLR, 2017).

[46] Akinc, A. et al. A combinatorial library of lipid-like materials for delivery of RNAi therapeutics. *Nat. Biotechnol.* **26**, 561–569 (2008). 18438401 10.1038/nbt1402 PMC3014085

[47] Whitehead, K. A. et al. Degradable lipid nanoparticles with predictable in vivo siRNA delivery activity. *Nat. Commun.* **5**, 4277 (2014). 24969323 10.1038/ncomms5277 PMC4111939

[48] Jiang, A. Y. et al. Combinatorial development of nebulized mRNA delivery formulations for the lungs. *Nat. Nanotechnol.* **19**, 364–375 (2024). 37985700 10.1038/s41565-023-01548-3 PMC10954414

[49] Paunovska, K. et al. A direct comparison of in vitro and in vivo nucleic acid delivery mediated by hundreds of nanoparticles reveals a weak correlation. *Nano Lett.* **18**, 2148–2157 (2018). 29489381 10.1021/acs.nanolett.8b00432 PMC6054134

[50] Miao, L. et al. Delivery of mRNA vaccines with heterocyclic lipids increases anti-tumor efficacy by STING-mediated immune cell activation. *Nat. Biotechnol.* **37**, 1174–1185 (2019). 31570898 10.1038/s41587-019-0247-3

[51] Zhu, Y. et al. Multi-step screening of DNA/lipid nanoparticles and co-delivery with siRNA to enhance and prolong gene expression. *Nat. Commun.* **13**, 4282 (2022). 35879315 10.1038/s41467-022-31993-y PMC9310361

[52] Li, B. et al. Combinatorial design of nanoparticles for pulmonary mRNA delivery and genome editing. *Nat. Biotechnol.* **41**, 1410–1415 (2023). 36997680 10.1038/s41587-023-01679-x PMC10544676

[53] Shuaibi, M. et al. Rotation invariant graph neural networks using spin convolutions. Preprint at https://arxiv.org/abs/2106.09575 (2021).

[54] Devlin, J., Chang, M.-W., Lee, K. & Toutanova, K. BERT: pre-training of deep bidirectional transformers for language understanding. In *Proc. NAACL-HLT* Vol. 1, 2 (2019).

[55] Xiong, R. et al. On layer normalization in the transformer architecture. In *International Conference on Machine Learning* 10524–10533 (PMLR, 2020).

[56] Ouyang, L. et al. Training language models to follow instructions with human feedback. *Adv. Neural Inf. Process. Syst.* **35**, 27730–27744 (2022).

[57] Touvron, H. et al. Llama 2: open foundation and fine-tuned chat models. Preprint at https://arxiv.org/abs/2307.09288 (2023).

[58] Whitehead, K. A. et al. Synergistic silencing: combinations of lipid-like materials for efficacious siRNA delivery. *Mol. Ther.* **19**, 1688–1694 (2011). 21750531 10.1038/mt.2011.141 PMC3182356

