# Supplementary material for: Designing lipid nanoparticles using a transformer-based neural network
Source: Nat Nanotechnol. 2025 Aug 15;20(10):1491–501. doi: 10.1038/s41565-025-01975-4 (PMC12534189; doi:10.1038/s41565-025-01975-4)
Supplement: Supplementary file 1 — Supplementary Figs. 1–22, discussion and Tables 1–23. [file 41565_2025_1975_MOESM1_ESM.pdf]

nature nanotechnology

Supplementary information https://doi.org/10.1038/s41565-025-01975-4

Designing lipid nanoparticles using a transformer-based neural network

In the format provided by the authors and unedited

Appendix A Supplementary Material

A.1 Interaction between PBAE and other LNP Features

To analyze interaction between PBAE and the other LNP features, we performed modeling experiments by in-filling PBAE LNP efficacy data with COMET's prediction, by predicting efficacy of LNPs with 5% interval of PBAE:ionizable lipid molar ratio to study following questions:

Q1) Do optimal PBAE choices depend on different cell types: Examining the three top-performing PBAE choices in PBAE LNPs for DC2.4 and B16-F10 and the PBAE (Table S1), we observe that the best PBAE polymers are different for these cell types. However, we also observe that some PBAEs such as A1D1-B1 and A1D1-B3 are amongst the top 3 PBAE choices for these two cell types, suggesting that there are PBAEs that are generally effective for these two cell types.

Q2) Do optimal PBAE % depend on different cell types: We observed that there are differences in the top-performing PBAE molar % and PBAE:IL molar ratio between the two cell lines. In DC2.4 cells, PBAE LNPs will require a higher PBAE content for higher efficacy ((Table S2 and S3), with PBAE:IL molar ratio around the value of 0.25 (Table S3). In contrast, for B16-F10 cells, PBAE LNPs will require a relatively lower PBAE content for higher efficacy, with PBAE:IL molar ratio around the value of 0.025 (Table S3), with exception for PBAEs that use the B2 branching agent.

Q3) Do optimal PBAE % depend on choice of ionizable lipid: To investigate this, we took PBAE LNP that has the highest predicted DC2.4 efficacy and extracted the PBAE molar % that gives the highest DC2.4 efficacy when its ionizable are substituted by other ionizable lipid choice, while keeping other PBAE and lipid variables (lipid choice and ratio) constant (Table S4 and S5). Interestingly, the optimal PBAE molar % across the three ionizable choices remains constant at a value of 0.0805 (Table S6).

Q4) Do optimal PBAE molar % depend on relative lipid ratios: To investigate this, we took PBAE LNP that has the highest predicted DC2.4 efficacy (containing P1 (A1D1-B1) PBAE) and extracted the PBAE molar % that gives the highest DC2.4 efficacy when the relative lipid ratio is different, while keeping other lipid variables constant (Table S7). We observed that the optimal PBAE molar % differs across the three lipid ratios, with the biggest difference when the helper lipid and cholesterol molar % changes (Table S8).

Table S1: PBAE polymers for top-performing LNPs in DC2.4 and B16-F10.

PBAE Rank	DC2.4		B16-F10	
	Monomer	Branching Agent	Monomer	Branching Agent
1	A1D1	B1	A2D1	B1
2	A1D3	B1	A1D1	B3
3	A1D1	B3	A1D1	B1

A.2 Interaction between Synergistic Ionizable Lipid Combinations and other LNP Features

To analyze interaction between PBAE and the other LNP features, we performed modeling experiments by predicting efficacy of synergistic ionizable lipid (IL) efficacy data with COMET's prediction by predicting efficacy of LNPs with 5% interval of IL_1:IL_2 ratio to study following questions:

Q1) Do choices of optimal IL pair depend on different cell types: We extracted the top performing synergistic ionizable pairs for each of the 13 major ionizable lipid in LANCE (Table S9) and observed that the optimal IL pair choices predominantly consist of two ionizable lipids (C12-200 and CKK-E12) for both DC2.4 and B16-F10. However, C12-200 is the dominant ionizable lipid for DC2.4-optimal LNPs while CKK-E12 is the dominant ionizable in B16-F10-optimal LNPs. Moreover, for certain ratios (e.g.

Table S2: Top three PBAE molar percentages (%) for each unique PBAE branching agent and PBAE monomer combination across two cell lines (DC2.4 and B16-F10).

PBAE Monomer	PBAE Branching Agent	DC2.4			B16-F10		
		1st	2nd	3rd	1st	2nd	3rd
A1D1	B1	8.05	7.15	9.50	0.87	1.04	1.21
A1D1	B2	9.50	8.05	10.91	9.50	10.91	12.28
A1D1	B3	8.05	7.15	9.50	0.87	1.04	1.21
A1D3	B1	8.05	9.50	7.15	0.87	1.04	1.21
A1D3	B2	9.50	10.91	8.05	9.50	10.91	12.28
A1D3	B3	8.05	7.15	9.50	0.87	1.04	8.05
A2D1	B1	8.05	9.50	7.15	0.87	1.04	1.21
A2D1	B2	9.50	10.91	8.05	0.87	1.04	1.21
A2D1	B3	8.05	9.50	7.15	0.87	1.04	1.21
A2D3	B1	9.50	8.05	10.91	0.87	1.04	1.21
A2D3	B2	9.50	10.91	12.28	9.50	10.91	12.28
A2D3	B3	8.05	9.50	10.91	0.87	1.04	8.05
A3D1	B1	6.24	5.30	7.15	0.87	1.04	1.21
A3D1	B2	6.24	7.15	5.30	0.87	1.04	1.21
A3D1	B3	6.24	5.30	7.15	0.87	1.04	1.21

35:8:54.5:2.5 and 35:28:34.5:2.5), the synergistic ionizable lipid pairs contain different ionizable lipids, suggesting that the optimal IL more heavily depends on cell type for certain lipid ratios.

Q2) Do choices of optimal IL pair depend on different lipid ratio: Lipid ratios with very high or very low helper lipid molar % (lower half of Table S9) have different optimal ionizable lipid pairs than other ratios (top half of Table S9), for both DC2.4 and B16-F10 cells, implying interaction between the lipid ratio and choice of synergistic ionizable lipid choices.

Q3) Do choices of optimal IL_1:IL_2 % depend on cell types: For the Ionizable Lipid : Helper Lipid : Cholesterol : PEG-Lipid ratio of '35:16:46.5:2.5', though the ionizable lipid pairs for both DC2.4 and B16-F10 contain the same ionizable lipids (C12-200 and CKK-E12), we observe that the optimal relative ratio between these two ionizable lipids are different, with C12-200 being the dominant ionizable lipid for DC2.4 and CKK-E12 being the dominant ionizable lipid for B16-F10 (Table S10).

Q4) Do optimal ionizable lipid pairs and IL_1:IL_2 molar % depend on relative lipid ratio: Similar to what we observed in Q6, lipid ratios with very high or very low helper lipid molar % (lower half of Table S11) have different optimal ionizable lipid pairs than other ratios (top half of Table S11), when the helper lipid, sterol, and PEG lipid are fixed as DOPE, Cholesterol and C14-PEG respectively. The optimal IL_1:IL_2 ratio for each IL_1/IL_2 pairs are rather consistent, with the exception of C12-200/SM-102 for DC2.4 where there is slight variation (2.33 to 9).

Q5) Do optimal ionizable lipid pairs and IL_1:IL_2 molar % depend on choices of other lipid types: We observed that as the helper lipid changes between DOPE and DSPC, the optimal IL_1/IL_2 pair and their relative ratio (IL_1:IL_2 Ratio) changes, for both DC2.4 and B16F10 (Table S12). In contrast, as

Table S3: Top three PBAE:IL ratios for each unique PBAE branching agent and PBAE monomer combination across two cell lines (DC2.4 and B16-F10).

Monomer	Branching Agent	DC2.4			B16-F10		
		1	2	3	1	2	3
A1D1	B1	0.250	0.220	0.300	0.025	0.030	0.035
A1D1	B2	0.300	0.250	0.350	0.300	0.350	0.400
A1D1	B3	0.250	0.220	0.300	0.025	0.030	0.035
A1D3	B1	0.250	0.300	0.220	0.025	0.030	0.035
A1D3	B2	0.300	0.350	0.250	0.300	0.350	0.400
A1D3	B3	0.250	0.220	0.300	0.025	0.030	0.250
A2D1	B1	0.250	0.300	0.220	0.025	0.030	0.035
A2D1	B2	0.300	0.350	0.250	0.025	0.030	0.035
A2D1	B3	0.250	0.300	0.220	0.025	0.030	0.035
A2D3	B1	0.300	0.250	0.350	0.025	0.030	0.035
A2D3	B2	0.300	0.350	0.400	0.300	0.350	0.400
A2D3	B3	0.250	0.300	0.350	0.025	0.030	0.250
A3D1	B1	0.190	0.160	0.220	0.025	0.030	0.035
A3D1	B2	0.190	0.220	0.160	0.025	0.030	0.035
A3D1	B3	0.190	0.160	0.220	0.025	0.030	0.035

Table S4: Lipid variables for PBAE % and ionizable lipid interaction analysis.

Lipid Component	Name	Lipid Molar Percentage (%), excluding PBAE
Helper lipid	DOPE	50
Sterol lipid	Cholesterol	12.5
PEG lipid	C14-PEG	2.5

Table S5: PBAE and ionizable lipid variable for PBAE % and ionizable lipid interaction analysis.

**Branching Agent**	B1
**Monomer**	A1D1
**Ionizable Lipid Molar Percentage (%), excluding PBAE**	35

Table S6: Optimal PBAE molar % for A1D1-B1 LNP with different ionizable lipid choices. The relative lipid ratios are the same across these three LNPs.

Ionizable Lipid	Optimal PBAE Molar Percentage (%) in PBAE LNP
SM-102	8.05
DLin-MC3-DMA	8.05
C12-200	8.05

Table S7: Lipid variables for PBAE percentage and lipid ratio interaction analysis.

Lipid Component	Name
Ionizable lipid	C12-200
Helper lipid	DOPE
Sterol lipid	Cholesterol
PEG lipid	C14-PEG

Table S8: Optimal PBAE molar percentage for A1D1-B1 LNP with different lipid ratios. The other lipid variables are the same across these three LNPs.

Lipid Ratio (Ionizable Lipid : Helper Lipid : Cholesterol : PEG-Lipid)	Optimal PBAE Molar Percentage
35:16:46.5:2.5 (Base)	0.0087
35:50:12.5:2.5 (C4)	0.0805
15:21.1:61.4:2.5 (I1)	0.0037

the sterol changes between cholesterol and Beta-sitosterol, the choice of ionizable lipid stays the same, except for one case ('DOPE, cholesterol' versus 'DOPE, Beta-sitosterol' for DC2.4) where the dominant and minor ionizable lipids are swapped.

Table S9: Optimal synergistic ionizable lipid pair for each cell line (DC2.4 and B16-F10), given selected lipid molar ratios. The more dominant lipid in terms of molar percentage is labeled as IL_1.

Lipid Ratio (Ionizable Lipid : Helper Lipid : Cholesterol : PEG-Lipid)	DC2.4		B16-F10	
	IL_1	IL_2	IL_1	IL_2
35:16:46.5:2.5	C12-200	CKK-E12	CKK-E12	C12-200
35.5:16.3:47.2:1	C12-200	CKK-E12	CKK-E12	C12-200
34.1:15.6:45.3:5	C12-200	CKK-E12	CKK-E12	C12-200
32.3:14.8:42.9:10	C12-200	CKK-E12	CKK-E12	C12-200
15:21.1:61.4:2.5	C12-200	CKK-E12	CKK-E12	C12-200
25:18.6:53.9:2.5	C12-200	CKK-E12	CKK-E12	C12-200
45:13.4:39.1:2.5	C12-200	CKK-E12	CKK-E12	L-319
55:10.9:31.6:2.5	C12-200	SM-102	CKK-E12	C12-200
35:8:54.5:2.5	C12-200	SM-102	CKK-E12	L-319
35:28:34.5:2.5	C12-200	SM-102	CKK-E12	L-319
35:50:12.5:2.5	C12-200	SM-102	C12-200	SM-102
47.5:32.5:17.5:2.5	C12-200	SM-102	CKK-E12	C12-200
47.5:34:17.5:1	C12-200	SM-102	CKK-E12	C12-200

Table S10: Optimal relative molar ratio of ionizable lipid C12-200 and CKK-E12 mix in LNPs with 'Ionizable Lipid : Helper Lipid : Cholesterol : PEG-Lipid' ratio of '35:16:46.5:2.5'.

**Cell Line**	C12-200 (Molar % of IL)	CKK-E12 (Molar % of IL)
DC2.4	95	5
B16-F10	5	95

Table S13: 13 Lipid ratios used in the LANCE dataset and their lipid type molar percentages. Values are in %.

Ratio Name	Ionizable lipid	PEG-lipid	Cholesterol	Helper lipid
BASE	35	2.5	46.5	16
P1	35.5	1	47.2	16.3
P2	34.1	5	45.3	15.6
P3	32.3	10	42.9	14.8
I1	15	2.5	61.4	21.1
I2	25	2.5	53.9	18.6
I3	45	2.5	39.1	13.4
I4	55	2.5	31.6	10.9
C1	35	2.5	54.5	8
C2	35	2.5	34.5	28
C3	35	2.5	12.5	50
M1	47.5	2.5	17.5	32.5
M2	47.5	1	17.5	34

Table S11: Optimal ionizable lipid choice and relative ionizable lipid molar ratio for each cell line (DC2.4 and B16-F10), given selected lipid molar ratios. The helper lipid, sterol, and PEG lipid are fixed as DOPE, Cholesterol and C14-PEG respectively to disentangle the effect from choices of other lipid types.

Lipid Ratio	DC2.4		B16-F10	
	IL_1/IL_2 Pair	$IL_1:IL_2$ Ratio	IL_1/IL_2 Pair	$IL_1:IL_2$ Ratio
35:16:46.5:2.5	C12-200/ CKK-E12	19	CKK-E12/ C12-200	19
35.5:16.3:47.2:1	C12-200/ CKK-E12	19	CKK-E12/ C12-200	19
34.1:15.6:45.3:5	C12-200/ CKK-E12	19	CKK-E12/ C12-200	19
32.3:14.8:42.9:10	C12-200/ CKK-E12	19	CKK-E12/ C12-200	19
15:21.1:61.4:2.5	C12-200/ CKK-E12	19	CKK-E12/ C12-200	19
25:18.6:53.9:2.5	C12-200/ CKK-E12	19	CKK-E12/ C12-200	19
45:13.4:39.1:2.5	C12-200/ CKK-E12	2.33	CKK-E12/ L-319	4
55:10.9:31.6:2.5	C12-200/ SM-102	5.67	CKK-E12/ C12-200	1
35:8:54.5:2.5	C12-200/ SM-102	3	CKK-E12/ L-319	4
35:28:34.5:2.5	C12-200/ SM-102	5.67	CKK-E12/ L-319	4
35:50:12.5:2.5	C12-200/ SM-102	5.67	C12-200/ SM-102	19
47.5:32.5:17.5:2.5	C12-200/ SM-102	9.	C12-200/ MC3	5.67
47.5:34:17.5:1	C12-200/ SM-102	5.67	C12-200/ L-319	5.67

Table S12: Optimal ionizable lipid choice and relative ionizable lipid molar ratio for each cell line (DC2.4 and B16-F10), given different helper lipid and sterol choices, with C14-PEG fixed as the PEG lipid. The 'Ionizable Lipid : Helper Lipid : Cholesterol : PEG-Lipid' ratio is '35:16:46.5:2.5'.

Helper Lipid, Sterol Lipid	DC2.4		B16-F10	
	IL_1/IL_2 Pair	$IL_1:IL_2$ Ratio	IL Pair	$IL_1:IL_2$ Ratio
DOPE, Cholesterol	C12-200/ CKK-E12	19	CKK-E12/ C12-200	19
DOPE, Beta-sitosterol	CKK-E12/ C12-200	19	CKK-E12/ C12-200	19
DSPC, Cholesterol	C12-200/ SM-102	9	CKK-E12/ L-319	4
DSPC, Beta-sitosterol	C12-200/ SM-102	9	CKK-E12/ L-319	4

Table S14: Information of lipids used in the LANCE dataset, name, chemical structure, and SMILES representation.

Component Type	Name	Structure	SMILES
Ionizable Lipid	ALC-0315	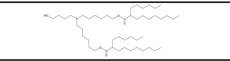	OCCCCN(CCCCCCOC(C(CCCCCC)CCCCCCCC)=O)CCCCCCOC(C(CCCCCC)CCCCCCCC)=O
	KC2	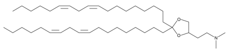	CCCCC/C=C\\C/C=C\\CCCCCCCCC1(OC(CCN(C)C)CO1)CCCCCCCC/C=C\\C/C=C\\CCCCC
	MC3	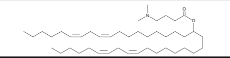	CCCCCC=CCC=CCCCCCCCCC(CCCCCCCCC=CCC=CCCCCC)OC(=O)CCCN(C)C
	SM-102	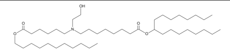	OCCN(CCCCCCCC(OC(CCCCCCCC)CCCCCCCC)=O)CCCCCC(OCCCCCCCCCCC)=O
	C12-200	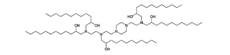	OC(CCCCCCCCCC)CN(CCN(CC(CCCCCCCCCC)O)CC(CCCCCCCCCC)O)CCN1CCN(CCN(CC(CCCCCCCCCC)O)CC(CCCCCCCCCC)O)CC1
	L319	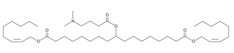	CN(C)CCCC(OC(CCCCCCCC(OC/C=C\\CCCCCC)=O)CCCCCCCC(OC/C=C\\CCCCCC)=O)=O
	CKK-E12	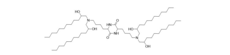	O=C1NC(CCCCN(CC(CCCCCCCCCC)O)CC(CCCCCCCCCC)O)C(NC1CCCCN(CC(CCCCCCCCCC)O)CC(CCCCCCCCCC)O)=O
Helper Lipid	DOPE	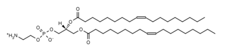	CCCCCCCCC=CCCCCCCCC(=O)OCC(COP(=O)(O)OCCN)OC(=O)CCCCCCCC=CCCCCCCCC
	DSPC	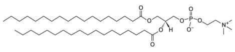	CCCCCCCCCCCCCCCCCC(=O)OCC(COP(=O)([O-])OCC[N+](C)(C)C)OC(=O)CCCCCCCCCCCCCCCCC
Sterol	Cholesterol	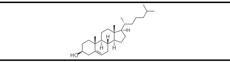	CC(C)CCCC(C)C1CCC2C1(CCC3C2CC=C4C3(CCC(C4)O)C)C
	DC-Cholesterol	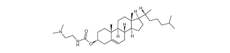	CC(C)CCCC(C)C1CCC2C1(CCC3C2CC=C4C3(CCC(C4)OC(=O)NCCN(C)C)C)C.Cl
	Beta-sitosterol	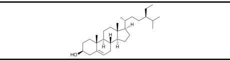	CCC(CCC(C)C1CCC2C1(CCC3C2CC=C4C3(CCC(C4)O)C)C)C(C)C
PEG-Lipid	C14-PEG	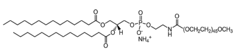	CCCCCCCCCCCCCC(=O)OCC(COP(=O)(O)OCCNC(=O)OCCOC)OC(=O)CCCCCCCCCCCCC.N
	C18-PEG	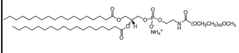	CCCCCCCCCCCCCCCCCC(=O)OCC(COP(=O)(O)OCCNC(=O)OCCOC)OC(=O)CCCCCCCCCCCCCCCCC.N

Algorithm S1 COMET Inference

function COMET($\{f_k\}, f^{\text{N/P\_ratio}}, f^{\text{phase\_ratio}}$):
  for $k = 1$ to $N_{\text{component}}$ **do**
    # Molecular structure embedding
    $z_k^{\text{molecule}} \leftarrow \text{MolecularEncoder}(f_k^{\text{molecule}})$
    # Molar percentage embedding
    $z_k^{\text{percent}} \leftarrow \text{GaussianLayer}_{\text{percent}}(f_k^{\text{molar\_percent}})$
    # Component type embedding
    $z_k^{\text{type}} \leftarrow \text{OneHotEncoding}_{\text{type}}(f_k^{\text{compound\_type}})$
    $z_k = \text{concat}(z_k^{\text{molecule}}, z_k^{\text{percent}}, z_k^{\text{type}})$
    $z_k \leftarrow \text{MLP}_{\text{project}}(z_k)$
  end for
  # Encode formulation-wide features
  $z_{\text{N/P}} \leftarrow \text{GaussianLayer}_{\text{N/P}}(f^{\text{N/P\_ratio}})$
  $z_{\text{phase}} \leftarrow \text{OneHotEncoding}_{\text{phase}}(f^{\text{phase\_ratio}})$
  # COMET's transformer inference
  for $l = 1$ to N_{block} **do**
    $z_{\text{CLS}}, \{z_k\}, z_{\text{N/P}}, z_{\text{phase}} \leftarrow \text{TransformerBlock}_l(z_{\text{CLS}}, \{z_k\}, z_{\text{N/P}}, z_{\text{phase}})$
  end for
  # Efficacy prediction with CLS token
  $y' \leftarrow \text{MLP}_{\text{predict}}(z_{\text{CLS}})$
  return y'
end function

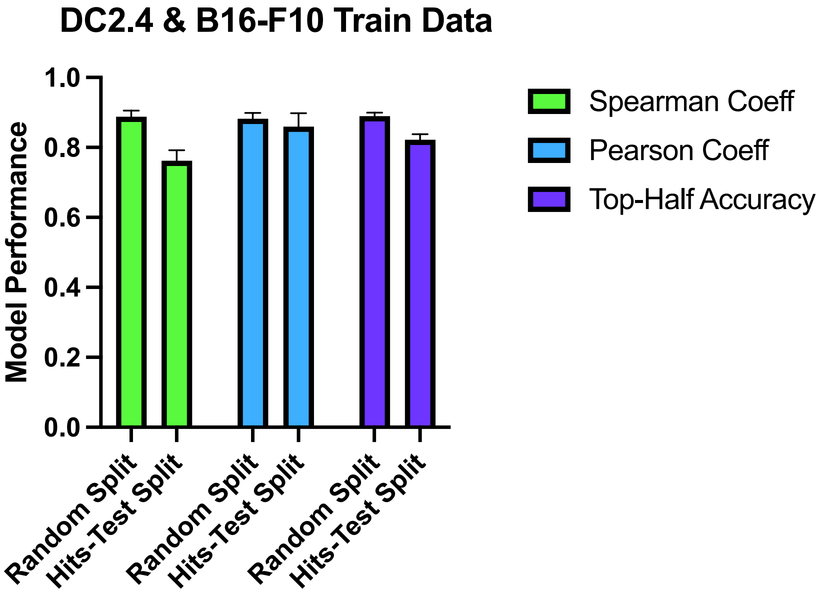

Fig. S1: Performance of COMET on different DC2.4 test data splits after training on both DC2.4 and B16-F10 data. Error bars are SEM. 20 replicates of training run with different random seeds were used for evaluation.

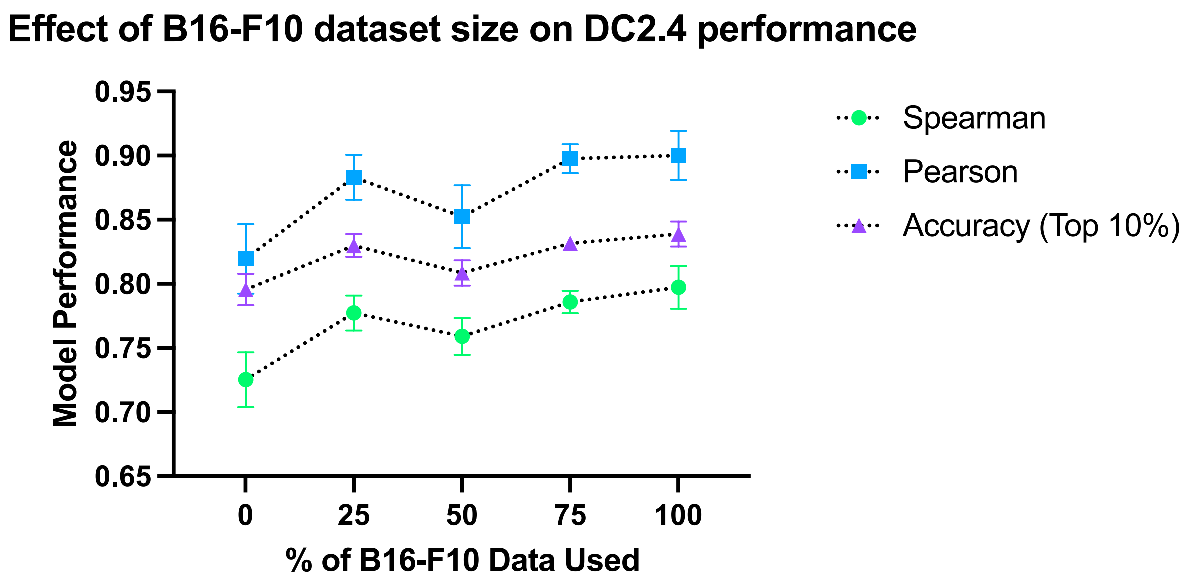

Fig. S2: Effect of B16-F10 dataset on the performance of COMET on DC2.4 efficacy prediction in the multitask learning setting. Error bars are SEM. 20 replicates of training run with different random seeds were used for evaluation.

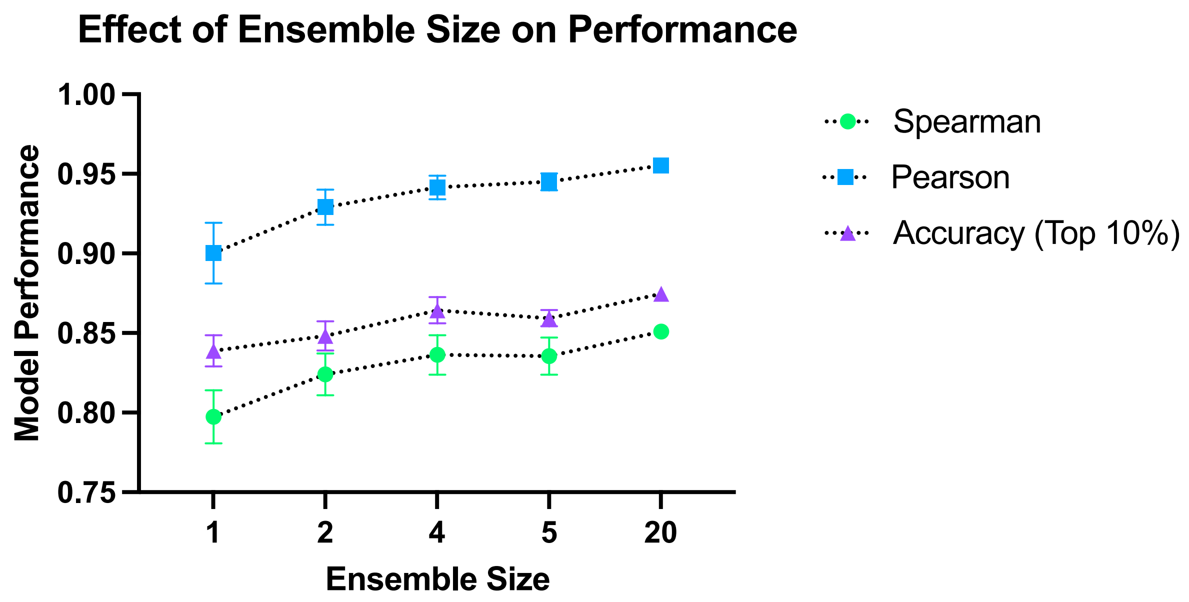

Fig. S3: Effect of ensemble size on the performance of COMET on DC2.4 efficacy prediction. Error bars are SEM. 20 replicates of model with different random seeds were used for evaluation.

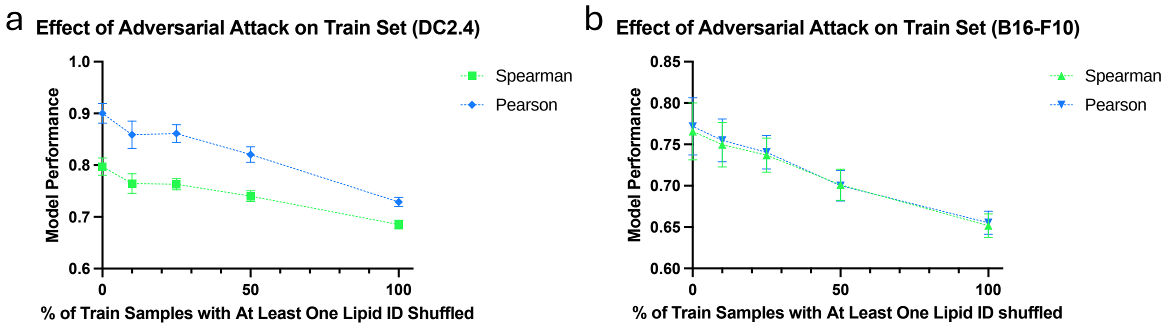

Fig. S4: Effect of material identity corruption on COMET's performance on (a) DC2.4 and (b) B16-F10 'hit-test' split. The x-axes show the proportion of training samples that has at least one lipid randomly substituted with a different lipid within the same lipid type. Error bars are SEM. 20 replicates of training run with different random seeds were used for evaluation.

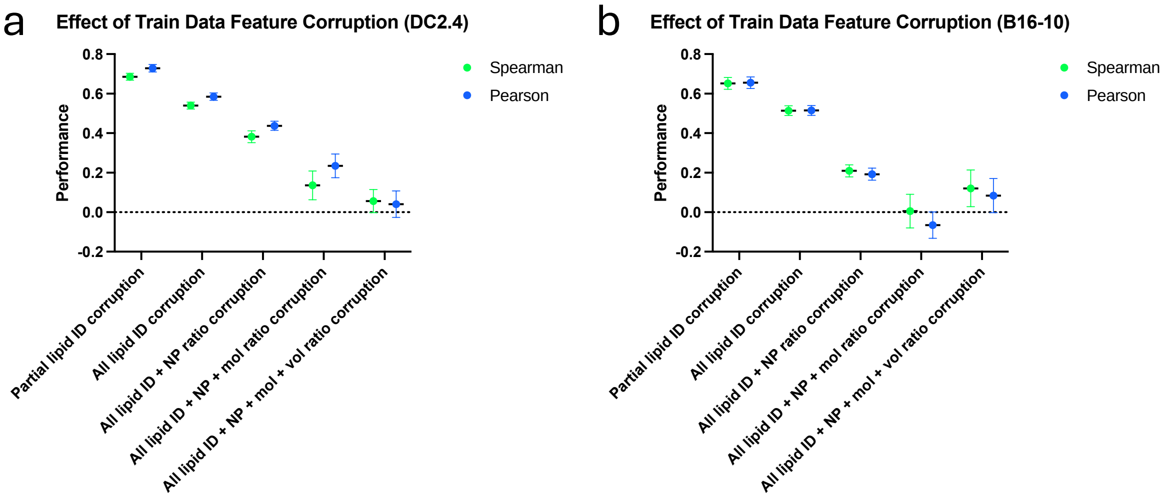

Fig. S5: Effect of train data corruption on COMET's performance on (a) DC2.4 and (b) B16-F10 'hit-test' split. The x-axes show the different train data feature corruptions applied to all train samples. Error bars are 95% CI. 20 replicates of training run with different random seeds were used for evaluation.

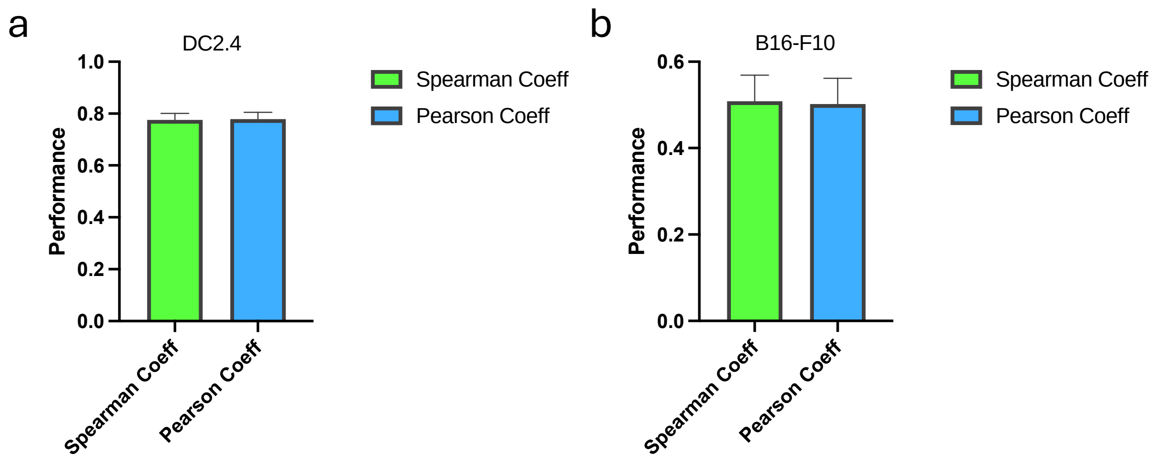

Fig. S6: Performance of COMET on test set with unseen lipid material (3 ionizable lipids: MC3, SM-102, CKK-E12 and a sterol: beta-sitosterol) on (a) DC2.4 and (b) B16-F10 efficacy. Error bars are SEM. 20 replicates of training run with different random seeds were used for evaluation.

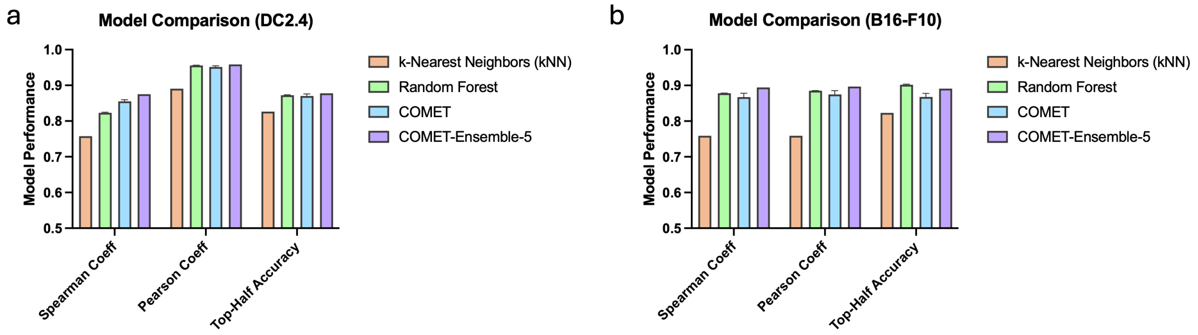

Fig. S7: Comparison of models' performance on (a) DC2.4 and (b) B16-F10 efficacy data on the 'hit-test' split. For Random Forest and COMET, top 5 models among 20 different (seed) initializations were used for comparison to remove effect from models that did not converge during training. Error bars are SEM.

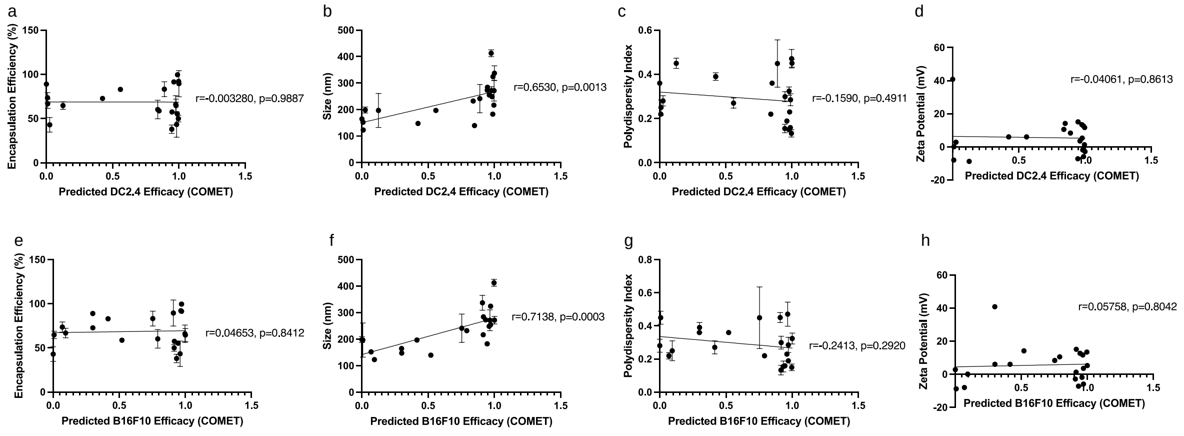

Fig. S8: Correlation (Pearson) between COMET prediction and key LNP properties for DC2.4 (a-d) and B16-F10 (e-h). COMET predicted values is normalized to fit the range between 0 and 1. Error bars are SEM.

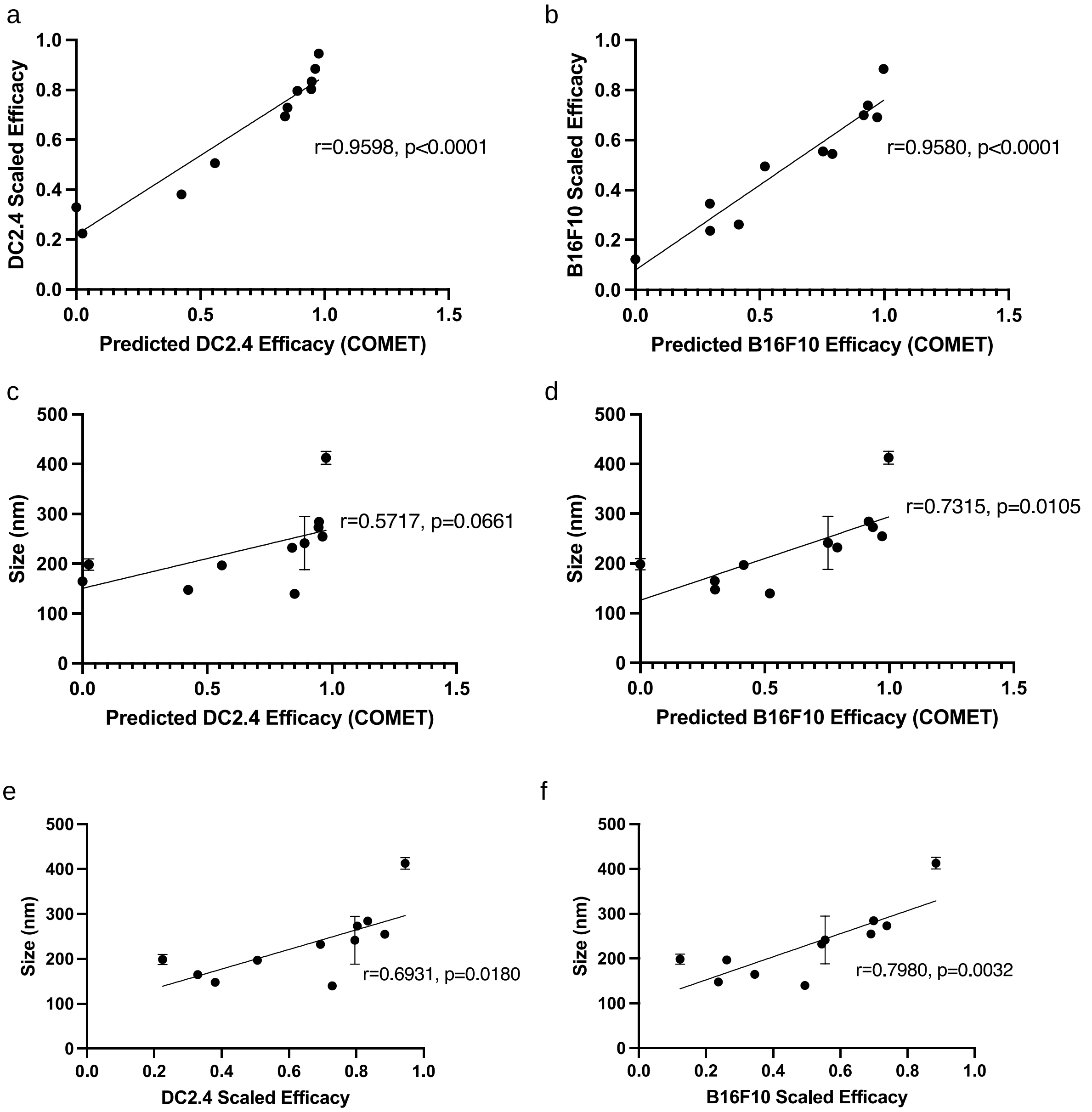

Fig. S9: (a-b) Correlation (Pearson) between COMET prediction and scaled efficacy values in DC2.4 and B16-F10 measured in vitro. (c-d) Correlation (Pearson) between COMET prediction and particle size for the same LNPs. COMET predicted values is normalized to fit the range between 0 and 1. (e-f) Correlation (Pearson) between particle size and scaled efficacy values in DC2.4 and B16-F10 measured in vitro. Error bars are SEM.

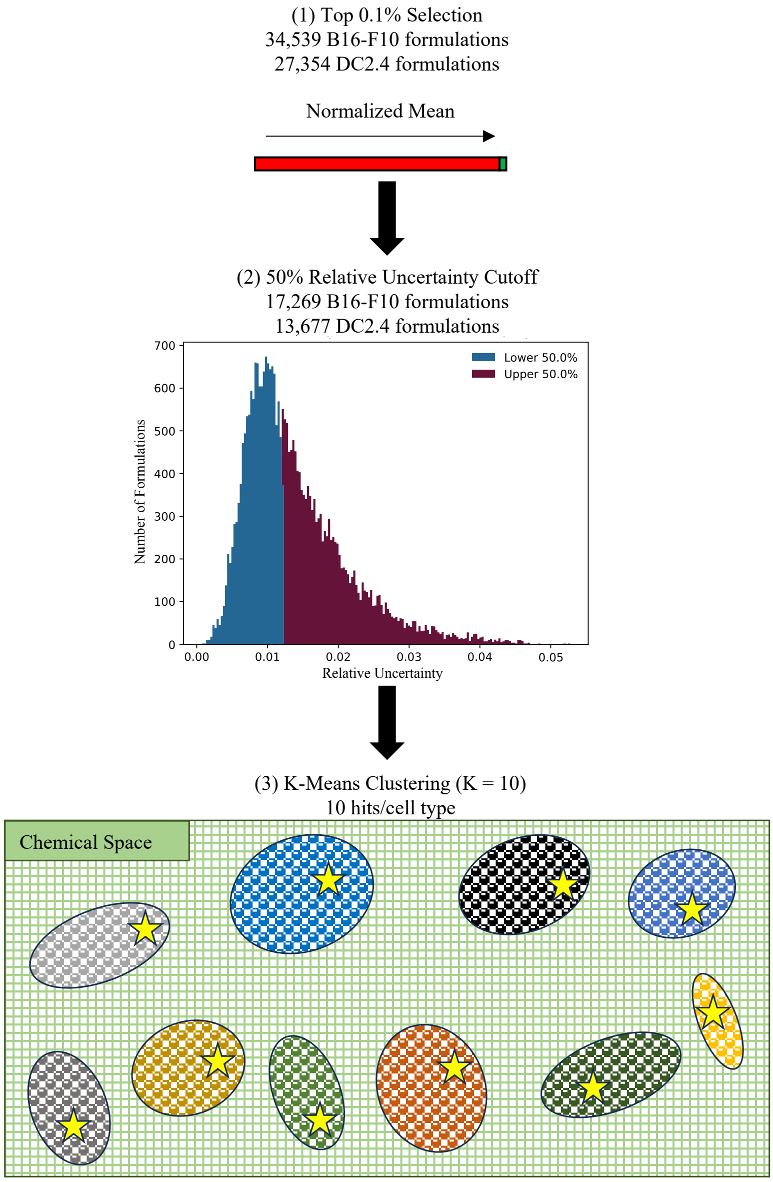

Fig. S10: **Exploratory Library Hit Screening Process**. Hits from the exploratory library are found by first selecting the top 0.1% scored candidates, removing formulations with highest-50% relative uncertainty values, then grouping them into chemically diverse clusters. The most highly scored candidate from each cluster (yellow stars) was selected as exploratory hits.

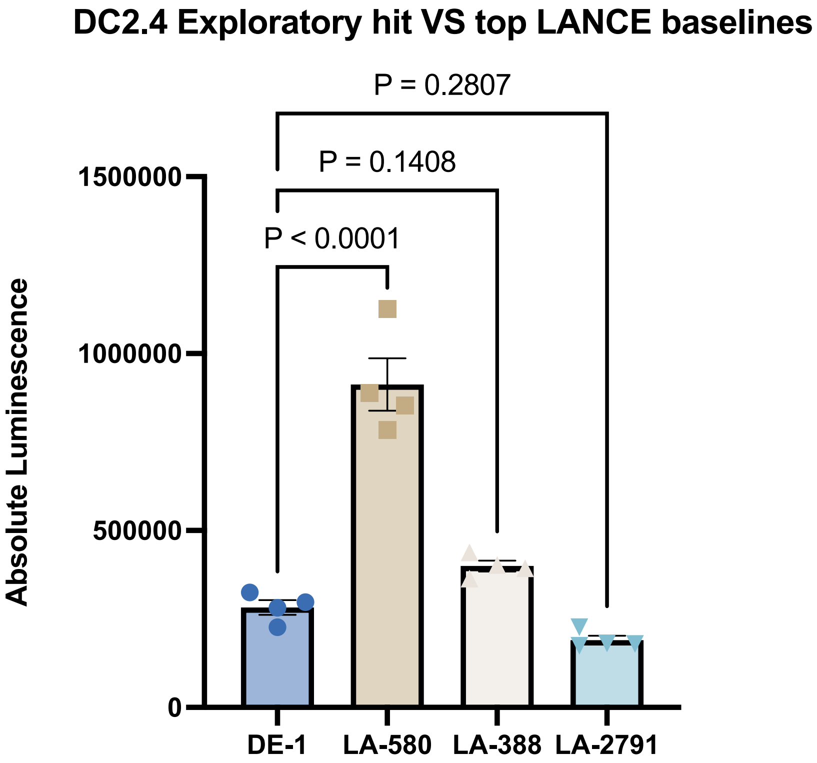

Fig. S11: Comparison between top COMET exploratory hit and top LANCE baseline LNPs for DC2.4 cells. Error bars are SEM. Statistical significances were determined using a one-way ANOVA with post hoc Tukey test.

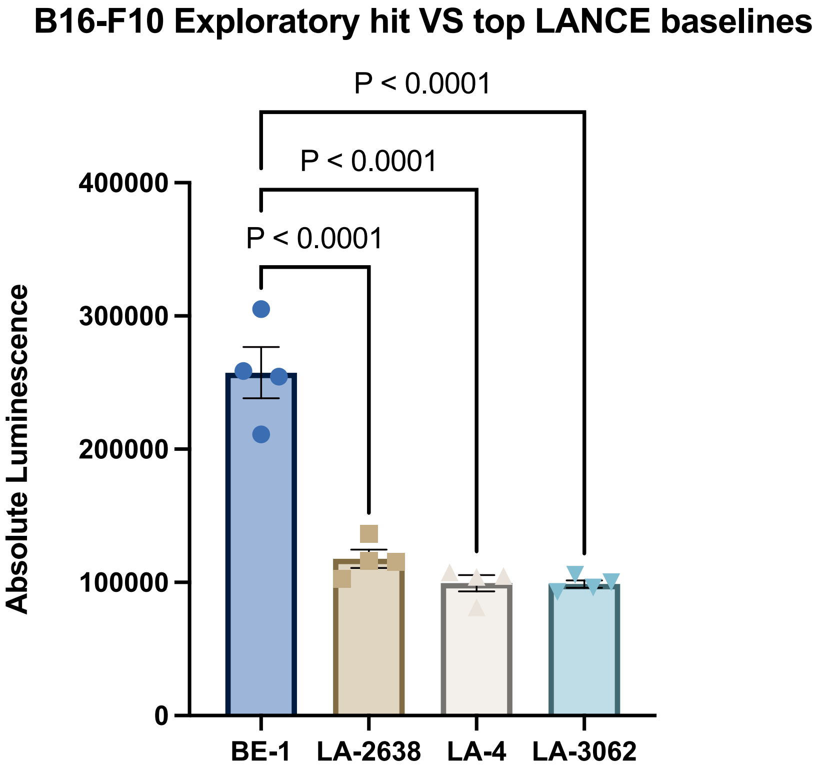

Fig. S12: Comparison between top COMET exploratory hit and top LANCE baseline LNPs for B16-F10 cells. Error bars are SEM. Statistical significances were determined using a one-way ANOVA with post hoc Tukey test.

Table S15: Lipid nanoparticle parameter ranges for exploratory libraries.

Exploratory Library Generation Parameters

LNP Parameter Type	LNP Parameter	Values
Molar Percentage	Ionizable Lipid	Min: 5%, Max: 85%, Step Size: 1%
	Helper Lipid	Min: 5%, Max: 85%, Step Size: 1%
	Cholesterol	Min: 5%, Max: 85%, Step Size: 1%
	PEG-Lipid	{ 1%, 2.5%, 5%, 10% }
Lipid Identities	Ionizable Lipid	{ ALC-0315, KC2, MC3, SM-102, C12-200, L319, CKK-E12 }
	Helper Lipid	{ DOPE, DSPC }
	Cholesterol	{ DC-Cholesterol, Cholesterol, Beta-Sitosterol }
	PEG-Lipid	{ C18-PEG, C14-PEG }
Synthesis Parameters	Ionizable Lipid Synergistic Ratio	{ 20%:80% , 40%:60% }
	Ionizable Lipid (MC3) : mRNA Weight Ratio	{ 10:1, 15:1, 20:1, 25:1, 30:1 }
	Volumetric Ratio	{ 1:1, 3:1 }

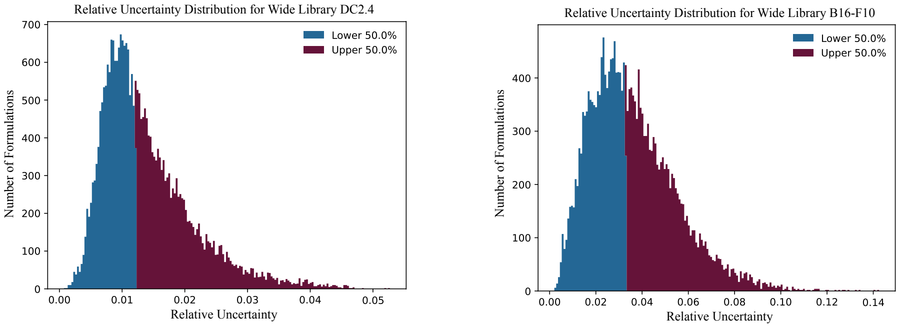

Fig. S13: Distribution of relative uncertainty values for exploratory DC2.4 (left) and B16-F10 (right) candidates.

Table S16: Name prefixes of LNPs and their meanings.

Prefix Naming Convention

Prefix	Cell Type	Library Type	Contains PBAE
BE	B16-F10	Exploratory	No
BO		Lead Optimization	
BOP			Yes
DE	DC2.4	Exploratory	No
DO		Lead Optimization	
DOP			Yes
LA	Both	LANCE	No
LAP			Yes

Table S17: Composition of COMET exploratory hits for DC2.4 cells.

DC2.4 Exploratory In-Silico Hits

Formulation ID	Ionizable Lipid(s)	Helper Lipid	Cholesterol(s)	PEG-Lipid(s)	N/P Ratio	Volumetric Ratio
DE-1	8.00% SM-102<br>12.0% CKK-E12	20.3% DOPE	58.7% Beta-Sitosterol	1.00% C14-PEG	12.7	1:1
DE-2	9.20% L319<br>13.8% CKK-E12	15.5% DOPE	60.5% Cholesterol	1.00% C14-PEG	14.8	1:1
DE-3	4.20% SM-102<br>16.8% CKK-E12	20.0% DOPE	58.0% Cholesterol	1.00% C14-PEG	12.7	1:1
DE-4	8.40% SM-102<br>12.6% CKK-E12	20.0% DOPE	58.0% Cholesterol	1.00% C14-PEG	12.7	1:1
DE-5	19.4% SM-102<br>12.9% CKK-E12	47.2% DOPE	10.5% Cholesterol	10.0% C14-PEG	18.5	1:1
DE-6	12.9% SM-102<br>19.4% CKK-E12	48.2% DOPE	9.50% Cholesterol	10.0% C14-PEG	16.9	1:1
DE-7	12.9% L319<br>19.4% CKK-E12	46.2% DOPE	11.5% Cholesterol	10.0% C14-PEG	16.9	1:1
DE-8	12.9% SM-102<br>19.4% CKK-E12	46.2% DOPE	11.5% Beta-Sitosterol	10.0% C14-PEG	21.2	1:1
DE-9	12.9% SM-102<br>19.4% CKK-E12	42.2% DOPE	15.5% Cholesterol	10.0% C14-PEG	21.2	1:1
DE-10	12.9% MC3<br>19.4% C12-200	48.2% DOPE	9.50% Cholesterol	10.0% C14-PEG	27.0	1:1

Table S18: Composition of COMET exploratory hits for B16-F10 cells.

B16-F10 Exploratory In-Silico Hits

Formulation ID	Ionizable Lipid(s)	Helper Lipid	Cholesterol(s)	PEG-Lipid(s)	N/P Ratio	Volumetric Ratio
BE-1	7.10% L319<br>28.4% CKK-E12	22.0% DOPE	41.5% Cholesterol	1.00% C14-PEG	14.3	1:1
BE-2	7.60% C12-200<br>11.4% CKK-E12	15.5% DOPE	64.5% Cholesterol	1.00% C14-PEG	16.9	1:1
BE-3	14.2% KC2<br>21.3% C12-200	22.0% DOPE	41.5% Cholesterol	1.00% C14-PEG	18.0	1:1
BE-4	10.0% L319<br>15.0% CKK-E12	14.0% DOPE	60.0% Cholesterol	1.00% C14-PEG	8.46	1:1
BE-5	12.6% C12-200<br>8.40% CKK-E12	18.0% DOPE	60.0% Beta-Sitosterol	1.00% C14-PEG	20.1	1:1
BE-6	9.60% KC2<br>14.4% CKK-E12	15.3% DOPE	59.7% Beta-Sitosterol	1.00% C14-PEG	8.46	1:1
BE-7	11.2% KC2<br>16.8% CKK-E12	13.2% DOPE	57.8% Cholesterol	1.00% C14-PEG	8.46	1:1
BE-8	18.0% C12-200<br>12.0% L319	13.7% DOPE	55.3% Cholesterol	1.00% C14-PEG	18.0	1:1
BE-9	13.6% KC2<br>20.4% C12-200	12.7% DOPE	52.3% Cholesterol	1.00% C14-PEG	18.0	1:1
BE-10	11.6% SM-102<br>17.4% C12-200	14.0% DOPE	56.0% Cholesterol	1.00% C14-PEG	18.0	1:1

Table S19: Composition of COMET lead optimization hits for DC2.4 cells, preceded by the original LANCE hit (e.g., LA-*).

DC2.4 Lead Optimization In-Silico Hits

Formulation ID	Ionizable Lipid(s)	Helper Lipid	Cholesterol(s)	PEG-Lipid(s)	N/P Ratio	Volumetric Ratio
LA-388	21.0% SM-102<br>14.0% CKK-E12	16.0% DOPE	46.5% Cholesterol	2.50% C14-PEG	7.40	1:1
DO-388-1	10.8% SM-102<br>16.2% CKK-E12	21.0% DOPE	50.5% Cholesterol	1.50% C14-PEG	8.46	1:1
DO-388-2	10.8% MC3<br>16.2% CKK-E12	22.0% DOPE	49.5% Cholesterol	1.50% C14-PEG	8.46	1:1
DO-388-3	11.2% L319<br>16.8% CKK-E12	16.0% DOPE	54.5% Cholesterol	1.50% C14-PEG	12.7	1:1
LA-580	10.0% L319<br>15.0% CKK-E12	18.6% DOPE	53.9% Cholesterol	2.50% C14-PEG	8.46	1:1
DO-580-1	6.80% L319<br>10.2% CKK-E12	27.6% DOPE	53.9% Cholesterol	1.50% C14-PEG	8.46	1:1
DO-580-2	9.20% SM-102<br>13.8% CKK-E12	20.6% DOPE	54.9% Cholesterol	1.50% C14-PEG	8.46	1:1
DO-580-3	9.20% SM-102<br>13.8% CKK-E12	19.6% DOPE	55.9% Cholesterol	1.50% C14-PEG	12.7	1:1
LA-2791	26.5% C12-200	36.4% DOPE	35.2% Cholesterol	1.89% C14-PEG	26.4	1:1
DO-2791-1	16.5% C12-200	44.4% DOPE	37.2% Cholesterol	1.89% C14-PEG	26.4	1:1
DO-2791-2	16.5% CKK-E12	46.4% DOPE	35.2% Cholesterol	1.89% C14-PEG	10.6	1:1
DO-2791-3	16.5% CKK-E12	46.4% DOPE	35.2% Cholesterol	1.89% C14-PEG	18.5	1:1

Table S20: Composition of COMET lead optimization hits for B16-F10 cells, preceded by the original LANCE hit (e.g., LA-*).

B16-F10 Lead Optimization In-Silico Hits

Formulation ID	Ionizable Lipid(s)	Helper Lipid	Cholesterol(s)	PEG-Lipid(s)	N/P Ratio	Volumetric Ratio
LA-4	14.2% ALC-0315<br>21.3% C12-200	16.3% DOPE	47.2% Cholesterol	1.00% C14-PEG	18.0	1:1
BO-4-1	13.4% ALC-0315<br>20.1% C12-200	15.3% DOPE	50.2% Cholesterol	1.00% C14-PEG	18.0	1:1
BO-4-2	12.6% KC2<br>18.9% C12-200	13.3% DOPE	54.2% Beta-Sitosterol	1.00% C14-PEG	18.0	1:1
BO-4-3	15.8% ALC-0315<br>23.7% L319	13.3% DOPE	46.2% Cholesterol	1.00% C14-PEG	17.2	1:1
LA -2638	27.6% C12-200	33.7% DOPE	36.7% Cholesterol	1.97% C14-PEG	26.4	1:1
BO-2638-1	20.6% C12-200	43.7% DOPE	35.7% Cholesterol	1.97% C14-PEG	26.4	1:1
BO-2638-2	20.6% C12-200	42.7% DOPE	33.7% Cholesterol	1.97% C14-PEG	26.4	1:1
BO-2638-3	20.6% C12-200	41.7% DOPE	34.7% Cholesterol	1.97% C14-PEG	33.1	1:1
LA -3062	35.1% C12-200	50.2% DOPE	12.2% Cholesterol	2.50% C14-PEG	26.4	1:1
BO-3062-1	28.1% C12-200	48.2% DOPE	22.2% Cholesterol	1.50% C14-PEG	26.4	1:1
BO-3062-2	32.1% C12-200	57.2% DOPE	9.2% Cholesterol	1.50% C18-PEG	26.4	1:1
BO-3062-3	29.1% C12-200	47.2% DOPE	22.2% Cholesterol	1.50% C14-PEG	29.1	1:1

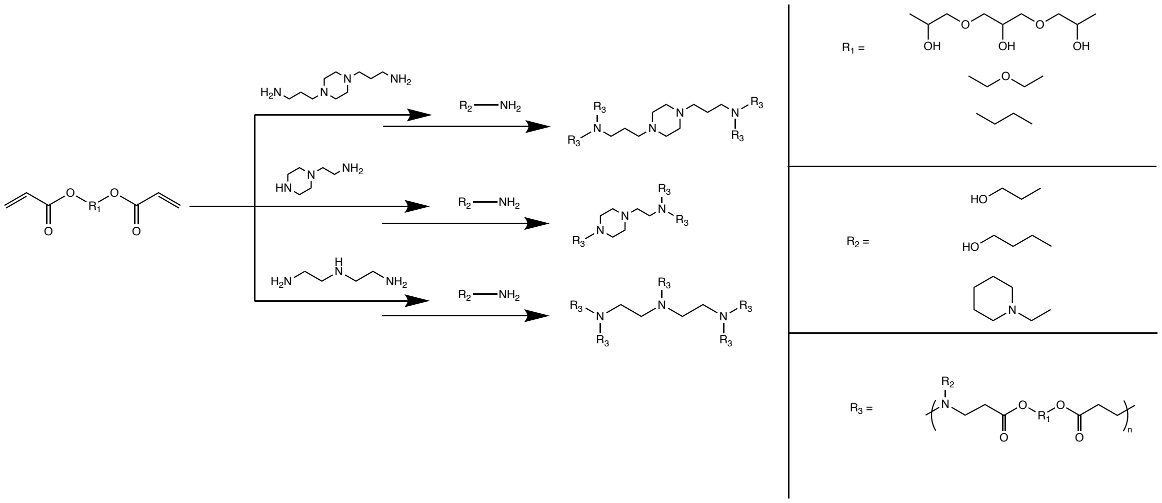

Fig. S14: Reaction scheme for the synthesis of PBAE polymers.

Table S21: Composition and names of amines, diacrylate and branching agent for PBAE synthesis. The code, A1, A2 and A3; D1, and D3; and B1, and B2 represents different amines, diacrylates and branching agents, respectively.

Polymer name	Amine	Diacrylate	Branching agent	Relative moles of branching agent	Relative moles of Amines	Relative moles of Diacrylate
**P1**	3-Amino-1-propanol (A1)	Glycerol 1,3-diglycerolate diacrylate (D1)	1,4-Bis(3-aminopropyl)piperazine (B1)	0.03	1	1
**P3**	3-Amino-1-propanol (A1)	1,4-Butanediol diacrylate (D3)	1,4-Bis(3-aminopropyl)piperazine (B1)	0.03	1	1
**P4**	3-Amino-1-propanol (A1)	Glycerol 1,3-diglycerolate diacrylate (D1)	1-(2-Aminoethyl)piperazine (B2)	0.05	1	1
**P6**	3-Amino-1-propanol (A1)	1,4-Butanediol diacrylate (D3)	1-(2-Aminoethyl)piperazine (B2)	0.05	1	1
**P7**	3-Amino-1-propanol (A1)	Glycerol 1,3-diglycerolate diacrylate (D1)	Diethylenetriamine (B3)	0.1	1	1
**P9**	3-Amino-1-propanol (A1)	1,4-Butanediol diacrylate (D3)	Diethylenetriamine (B3)	0.1	1	1
**P10**	5-amino-1-pentanol (A2)	Glycerol 1,3-diglycerolate diacrylate (D1)	1,4-Bis(3-aminopropyl)piperazine (B1)	0.03	1	1
**P12**	5-amino-1-pentanol (A2)	1,4-Butanediol diacrylate (D3)	1,4-Bis(3-aminopropyl)piperazine (B1)	0.03	1	1
**P13**	5-amino-1-pentanol (A2)	Glycerol 1,3-diglycerolate diacrylate (D1)	1-(2-Aminoethyl)piperazine (B2)	0.05	1	1
**P15**	5-amino-1-pentanol (A2)	1,4-Butanediol diacrylate (D3)	1-(2-Aminoethyl)piperazine (B2)	0.05	1	1
**P16**	5-amino-1-pentanol (A2)	Glycerol 1,3-diglycerolate diacrylate (D1)	Diethylenetriamine (B3)	0.1	1	1
**P18**	5-amino-1-pentanol (A2)	1,4-Butanediol diacrylate (D3)	Diethylenetriamine (B3)	0.1	1	1
**P19**	1-(2-Aminoethyl)piperidine (A3)	Glycerol 1,3-diglycerolate diacrylate (D1)	1,4-Bis(3-aminopropyl)piperazine (B1)	0.03	1	1
**P22**	1-(2-Aminoethyl)piperidine (A3)	Glycerol 1,3-diglycerolate diacrylate (D1)	1-(2-Aminoethyl)piperazine (B2)	0.05	1	1
**P25**	1-(2-Aminoethyl)piperidine (A3)	Glycerol 1,3-diglycerolate diacrylate (D1)	Diethylenetriamine (B3)	0.1	1	1

Table S22: Composition of COMET PBAE LNP lead optimization hits for DC2.4 cells, preceded by the original LANCE PBAE LNP hit (e.g., LAP-*).

DC2.4 PBAE Lead Optimization In-Silico Hits

Formulation ID	Ionizable Lipid(s)	Helper Lipid	Cholesterol(s)	PEG-Lipid(s)	PBAE Repeating Unit	PBAE Branching Agent	N/P Ratio	Volumetric Ratio
LAP-3234	32.2% C12-200	46.0% DOPE	11.5% Cholesterol	2.30% C14-PEG	8.05% A3D1	8.05% B2	26.4	1:1
DOP-3234-1	25.3% C12-200	55.3% DOPE	9.84% Cholesterol	3.28% C14-PEG	6.32% A3D1	6.32% B2	26.4	1:1
DOP-3234-2	23.5% C12-200	55.5% DOPE	11.8% Beta-Sitosterol	3.29% C14-PEG	4.88% A3D1	4.88% B2	26.4	1:1
DOP-3234-3	15.2% SM-102<br>10.1% C12-200	56.2% DOPE	9.84% Cholesterol	2.34% C14-PEG	6.32% A3D1	6.32% B2	13.7	1:1
LAP-3536	14.5% C12-200	20.3% DOPE	59.2% Cholesterol	2.41% C14-PEG	3.61% A3D1	3.61% B3	26.4	1:1
DOP-3536-1	5.91% C12-200	30.6% DOPE	60.5% Cholesterol	1.48% C14-PEG	1.48% A3D1	1.48% B3	26.4	1:1
DOP-3536-2	10.7% CKK-E12	30.3% DOPE	54.9% Cholesterol	1.46% C14-PEG	2.68% A3D1	2.68% B3	10.6	1:1
DOP-3536-3	2.40% SM-102<br>3.50% C12-200	30.6% DOPE	60.5% Cholesterol	1.48% C14-PEG	1.48% A3D1	1.48% B3	17.8	1:1

Table S23: Composition of COMET PBAE LNP lead optimization hits for B16-F10 cells, preceded by the original LANCE PBAE LNP hit (e.g., LAP-*).

B16-F10 PBAE Lead Optimization In-Silico Hits

Formulation ID	Ionizable Lipid(s)	Helper Lipid	Cholesterol(s)	PEG-Lipid(s)	PBAE Repeating Unit	PBAE Branching Agent	N/P Ratio	Volumetric Ratio
LAP-3234	32.2% C12-200	46.0% DOPE	11.5% Cholesterol	2.30% C14-PEG	8.05% A3D1	8.05% B2	26.4	1:1
BOP-3234-1	30.5% C12-200	44.3% DOPE	8.78% Cholesterol	8.78% C14-PEG	7.62% A3D1	7.62% B2	26.4	1:1
BOP-3234-2	28.8% CKK-E12	45.5% DOPE	9.74% Cholesterol	8.82% C14-PEG	7.19% A3D1	7.19% B2	10.6	1:1
BOP-3234-3	14.2% C12-200<br>21.3% CKK-E12	47.4% DOPE	5.92% Cholesterol	2.28% C14-PEG	8.88% A3D1	8.88% B2	16.9	1:1
LAP-3513	14.9% C12-200	20.9% DOPE	60.9% Cholesterol	2.48% C14-PEG	0.744% A3D1	0.744% B3	26.4	1:1
BOP-3513-1	16.9% C12-200	22.9% DOPE	57.9% Cholesterol	1.49% C14-PEG	0.843% A3D1	0.843% B3	26.4	1:1
BOP-3513-2	16.9% CKK-E12	22.9% DOPE	57.9% Cholesterol	1.49% C14-PEG	0.843% A3D1	0.843% B3	10.6	1:1
BOP-3513-3	12.5% C12-200<br>8.30% CKK-E12	21.9% DOPE	54.8% Cholesterol	1.48% C14-PEG	1.04% A3D1	1.04% B3	20.1	1:1

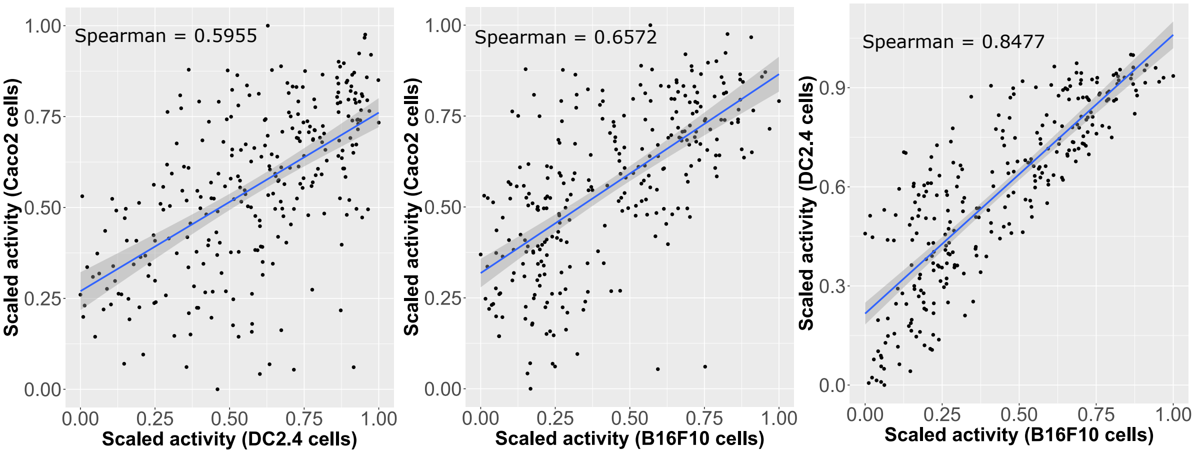

Fig. S15: Correlation between formulation activities in human intestinal epithelial Caco-2 cells, murine cancer B16-F10 cells and murine immune DC2.4 cells.

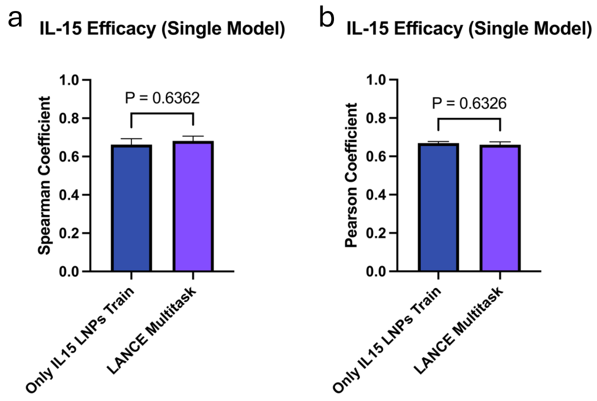

Fig. S16: Evaluation of COMET's performance, (a) Spearman rank coefficient and (b) Pearson coefficient, on predicting LNPs' efficacy in delivering IL-15 mRNA payload to HepG2, when evaluated as a single model. Statistical significances were determined using unpaired t-test. Error bars are SEM. 20 replicates of training run with different random seeds were used for evaluation.

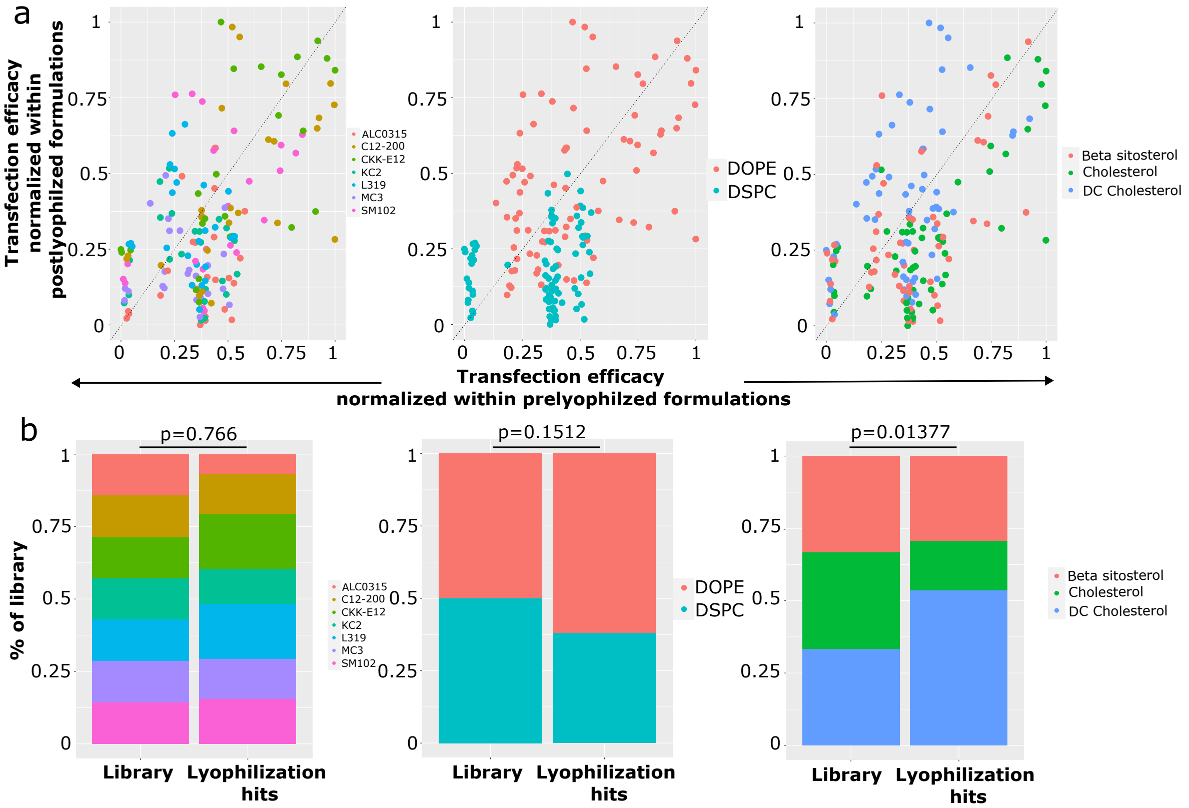

Fig. S17: (a) The dot plots show a measure of the activity of the LNPs before (x-axis) and after (y-axis) lyophilization. The color of the dots shows the composition of the LNPs. The activities of formulations were log-normalized to be scored 0-1 within the pre-lyophilization and post-lyophilization datasets. The dotted diagonal line is indicative of formulations that showed no change in their ranking after lyophilization. (b) The bar charts show the frequency of the lipid components in the whole library and in formulations that lie above the diagonal line in the dot plots in **a**. The formulations above the diagonal line are those formulations whose relative ranking improved after lyophilization. Statistics was determined with Pearson's Chi squared test.

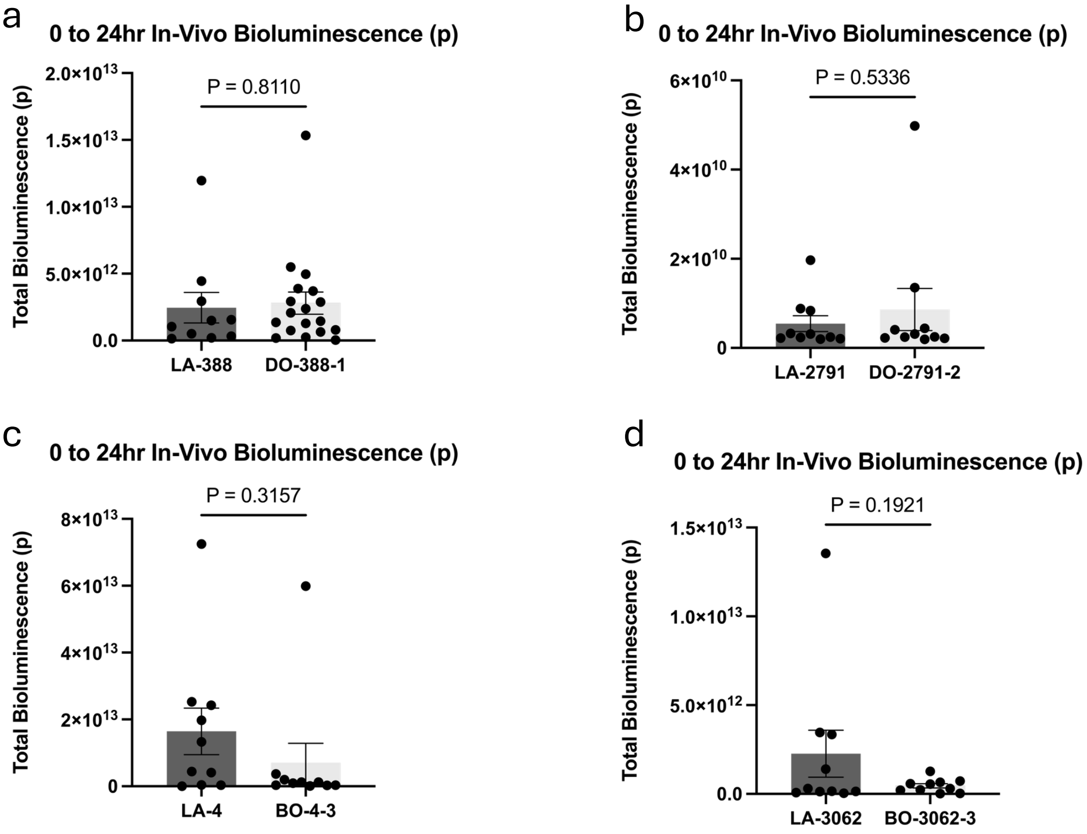

Fig. S18: Comparison of in-vivo efficacy between lead LNPs optimized by COMET (with prefix DO or BO) and their corresponding top-performing LANCE LNPs (with prefix LA) in subcutaneous administration of FLuc mRNA in mice. Error bars are SEM. Statistical significances were determined using unpaired two-tailed t-test.

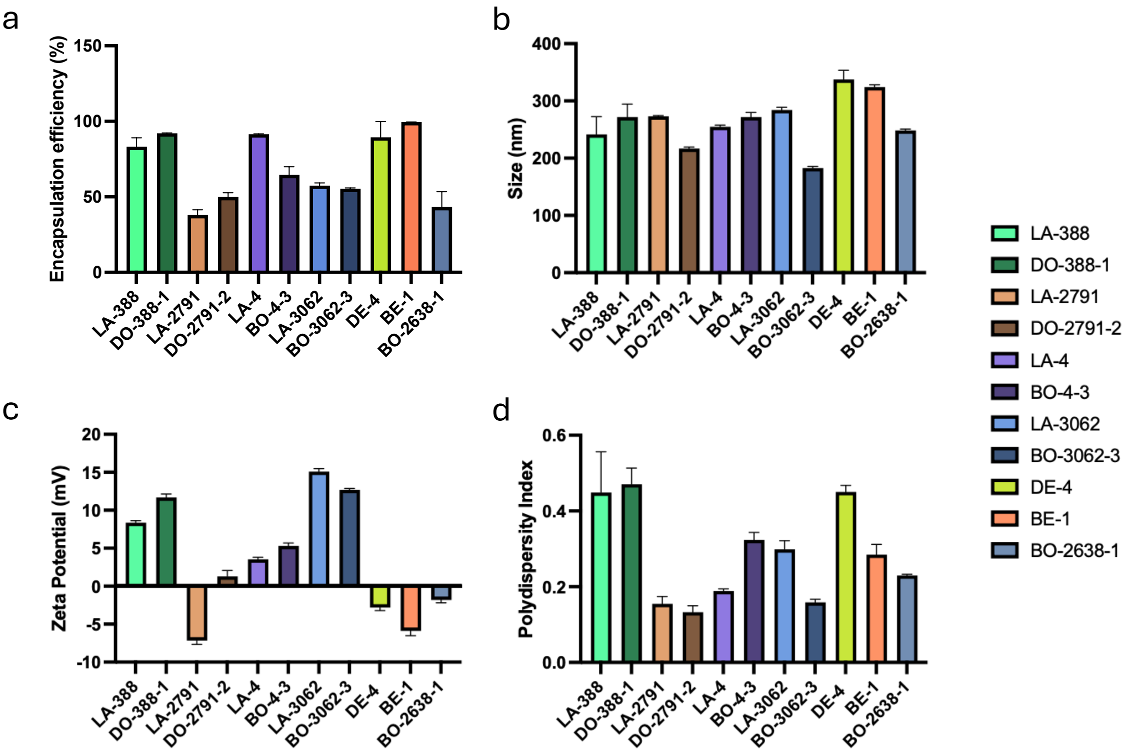

Fig. S19: Characterization of LNPs used in mouse studies. (a) mRNA encapsulation efficiency of LNPs, n = 2 technical replicates. (b) Size of LNPs, n = 3 technical replicates. (c) Zeta potential of LNPs, n = 3 technical replicates. (d) polydispersity index of LNPs, n = 3 technical replicates. Error bars are SEM.

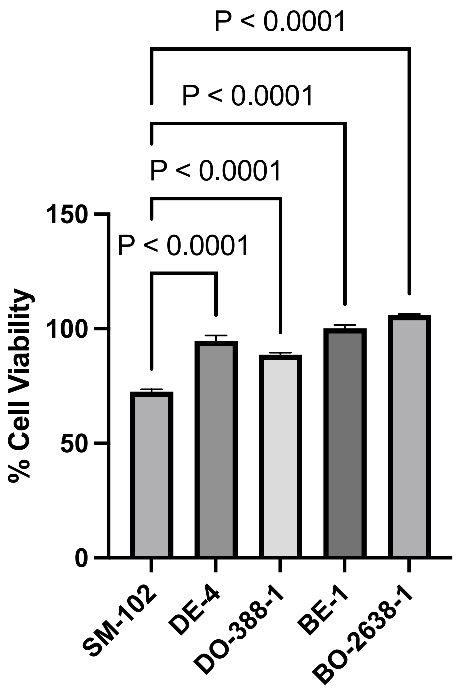

Fig. S20: Toxicity of in-vivo COMET hit LNPs measured via HepG2 viability. n = 4 technical replicates. Cells were seeded at 10K cells per well. Error bars are SEM. Statistical significances were determined using a one-way ANOVA with post hoc Dunnett test.

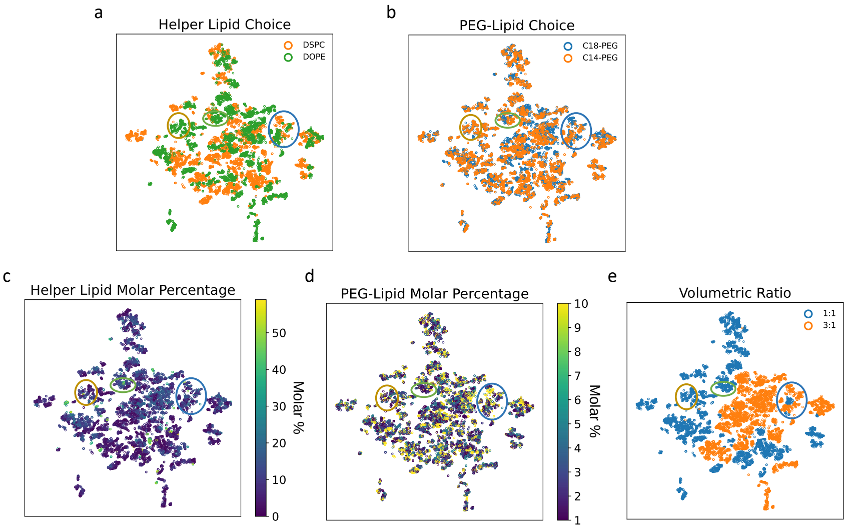

Fig. S21: (a-e) t-SNE visualization of COMET representations of 10K virtual LNPs, each sample colored by its predicted efficacy or feature value.

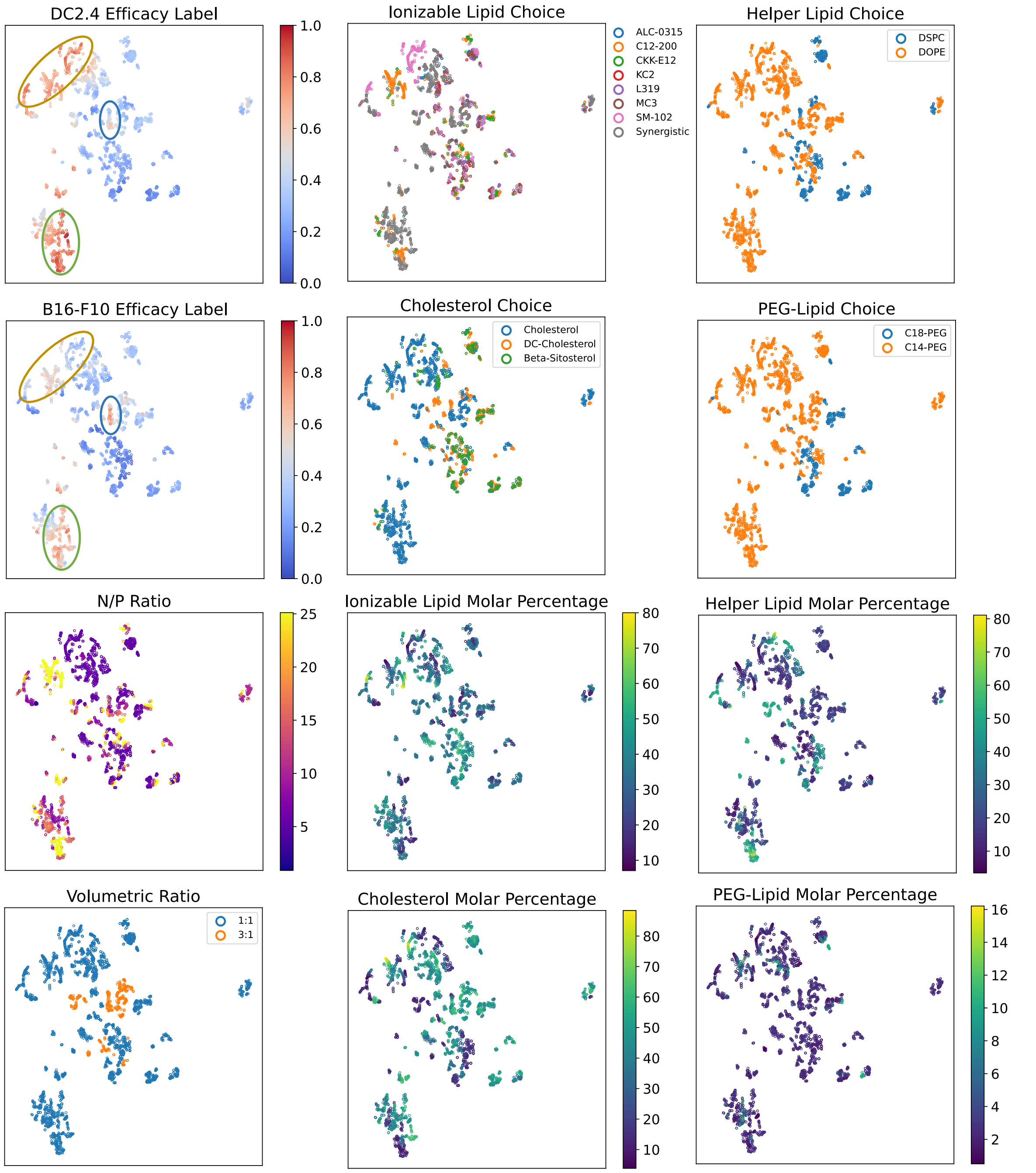

Fig. S22: t-SNE visualization of COMET representations of LANCE LNPs, each sample colored by its predicted efficacy or feature value.
