# Supplementary material for: Designing lipid nanoparticles using a transformer-based neural network
Source: Nat Nanotechnol. 2025 Aug 15;20(10):1491–501. doi: 10.1038/s41565-025-01975-4 (PMC12534189; doi:10.1038/s41565-025-01975-4)
Supplement: Supplementary file 2 — Reporting Summary [file 41565_2025_1975_MOESM2_ESM.pdf]

nature portfolio

Corresponding author(s): Chan; Kirtane; Traverso

Last updated by author(s): May 20, 2025

Reporting Summary

Nature Portfolio wishes to improve the reproducibility of the work that we publish. This form provides structure for consistency and transparency in reporting. For further information on Nature Portfolio policies, see our Editorial Policies and the Editorial Policy Checklist.

Statistics

For all statistical analyses, confirm that the following items are present in the figure legend, table legend, main text, or Methods section.

n/a	Confirmed	
☐	☒	The exact sample size (*n*) for each experimental group/condition, given as a discrete number and unit of measurement
☐	☒	A statement on whether measurements were taken from distinct samples or whether the same sample was measured repeatedly
☐	☒	The statistical test(s) used AND whether they are one- or two-sided<br>*Only common tests should be described solely by name; describe more complex techniques in the Methods section.*
☒	☐	A description of all covariates tested
☐	☒	A description of any assumptions or corrections, such as tests of normality and adjustment for multiple comparisons
☐	☒	A full description of the statistical parameters including central tendency (e.g. means) or other basic estimates (e.g. regression coefficient) AND variation (e.g. standard deviation) or associated estimates of uncertainty (e.g. confidence intervals)
☐	☒	For null hypothesis testing, the test statistic (e.g. *F*, *t*, *r*) with confidence intervals, effect sizes, degrees of freedom and *P* value noted<br>*Give P values as exact values whenever suitable.*
☒	☐	For Bayesian analysis, information on the choice of priors and Markov chain Monte Carlo settings
☒	☐	For hierarchical and complex designs, identification of the appropriate level for tests and full reporting of outcomes
☒	☐	Estimates of effect sizes (e.g. Cohen's *d*, Pearson's *r*), indicating how they were calculated

Our web collection on statistics for biologists contains articles on many of the points above.

Software and code

Policy information about availability of computer code

Data collection	Source code for the COMET model, trained weights and inference scripts will be made available at Github upon publication. The COMET model was developed with PyTorch (https://github.com/pytorch/pytorch).<br><br>t-SNE visualizations were generated with sckit-learn (https://scikit-learn.org). Integrated gradient analysis code was developed with Captum (https://captum.ai)<br><br>In-vitro bioluminescence data were collected with Agilent Biotek Cytation. In-vivo bioluminescence data were collected with IVIS Spectrum bioluminescent and fluorescent imaging systems (Perkin Elmer) and analyzed with Living Image software.
Data analysis	Data was analyzed using Python 3 and plotted using the Python Matplotlib library or GraphPad PRISM 10. Code is available on reasonable request from the corresponding author.

For manuscripts utilizing custom algorithms or software that are central to the research but not yet described in published literature, software must be made available to editors and reviewers. We strongly encourage code deposition in a community repository (e.g. GitHub). See the Nature Portfolio guidelines for submitting code & software for further information.

Data

Policy information about availability of data

All manuscripts must include a data availability statement. This statement should provide the following information, where applicable:

- Accession codes, unique identifiers, or web links for publicly available datasets
- A description of any restrictions on data availability
- For clinical datasets or third party data, please ensure that the statement adheres to our policy

The data supporting the findings of this study are available within the paper and its Supplementary Information files. Should any raw data files be needed in another format they are available from the corresponding author upon reasonable request.

Human research participants

Policy information about studies involving human research participants and Sex and Gender in Research.

Reporting on sex and gender	N/A
Population characteristics	N/A
Recruitment	N/A
Ethics oversight	N/A

Note that full information on the approval of the study protocol must also be provided in the manuscript.

Field-specific reporting

Please select the one below that is the best fit for your research. If you are not sure, read the appropriate sections before making your selection.

☒ Life sciences ☐ Behavioural & social sciences ☐ Ecological, evolutionary & environmental sciences

For a reference copy of the document with all sections, see nature.com/documents/nr-reporting-summary-flat.pdf

Life sciences study design

All studies must disclose on these points even when the disclosure is negative.

Sample size	Sample sizes were chosen based on preliminary experiments and prior work on lipid nanoparticles, to provide sufficient power for statistical comparison when needed.
Data exclusions	No data values were excluded in the analysis.
Replication	Experiment in general were repeated to confirm the results. All attempts at replication were successful.
Randomization	Cells from the same passages were randomly used for experiment groups or control groups. The results were confirmed by different cells l passages. Group allocation for mouse studies was performed randomly.
Blinding	While true blinding was not applied due to the proof-of-concept developmental nature of this study, data collection and analyses for cell and animal experiments were performed by separate individuals.

Reporting for specific materials, systems and methods

We require information from authors about some types of materials, experimental systems and methods used in many studies. Here, indicate whether each material, system or method listed is relevant to your study. If you are not sure if a list item applies to your research, read the appropriate section before selecting a response.

Materials & experimental systems

n/a	Involved in the study
☒	☐ Antibodies
☐	☒ Eukaryotic cell lines
☒	☐ Palaeontology and archaeology
☐	☒ Animals and other organisms
☒	☐ Clinical data
☒	☐ Dual use research of concern

Methods

n/a	Involved in the study
☒	☐ ChIP-seq
☒	☐ Flow cytometry
☒	☐ MRI-based neuroimaging

Eukaryotic cell lines

Policy information about cell lines and Sex and Gender in Research

Cell line source(s)	Cell lines were originally obtained from ATCC.
Authentication	The cell lines were not further authenticated after receiving from ATCC.
Mycoplasma contamination	Cell lines were tested negative for mycoplasma contamination.
Commonly misidentified lines (See ICLAC register)	The cell lines used in this article are not in the list of misidentified lines.

Animals and other research organisms

Policy information about studies involving animals; ARRIVE guidelines recommended for reporting animal research, and Sex and Gender in Research

Laboratory animals	Six-week-old female C57BL/6J mice (Strain #000664) from Jackson laboratory were used; A 14-hour light/10-hour dark cycle and a temperature of 65-75°F (~18-23°C) with 40-60% humidity are used. For each study, the mice involved were randomly assigned to treatment and control groups.
Wild animals	No wild animals were used in the study
Reporting on sex	Female
Field-collected samples	No field-collected samples were used in the study
Ethics oversight	This study is approved by MIT committee on Animal Care

Note that full information on the approval of the study protocol must also be provided in the manuscript.
